# Intelligent deep learning architecture for precision vegetable disease detection advancing agricultural new quality productive forces

**DOI:** 10.3389/fpls.2025.1611865

**Published:** 2025-08-13

**Authors:** Jun Liu, Xuewei Wang, Qian Chen, Peng Yan, Dugang Guo

**Affiliations:** ^1^ Shandong Provincial University Laboratory for Protected Horticulture, Weifang University of Science and Technology, Weifang, China; ^2^ School of Computer, Sichuan Technology and Business University, Chengdu, Sichuan, China; ^3^ The Industry-Education Integration Office, Sichuan Technology and Business University, Chengdu, Sichuan, China

**Keywords:** agricultural new quality productive forces, deep learning, vegetable disease detection, YOLO, precision agriculture, greenhouse cultivation, attention mechanism

## Abstract

In the context of advancing agricultural new quality productive forces, addressing the challenges of uneven illumination, target occlusion, and mixed infections in greenhouse vegetable disease detection becomes crucial for modern precision agriculture. To tackle these challenges, this study proposes YOLO-vegetable, a high-precision detection algorithm based on improved You Only Look Once version 10 (YOLOv10). The framework incorporates three innovative modules. The Adaptive Detail Enhancement Convolution (ADEConv) module employs dynamic parameter adjustment to preserve fine-grained features while maintaining computational efficiency. The Multi-granularity Feature Fusion Detection Layer (MFLayer) improves small target localization accuracy through cross-level feature interaction mechanisms. The Inter-layer Dynamic Fusion Pyramid Network (IDFNet) combines with Attention-guided Adaptive Feature Selection (AAFS) mechanism to enhance key information extraction capability. Experimental validation on our self-built Vegetable Disease Dataset (VDD, 15,000 images) demonstrates that YOLO-vegetable achieves 95.6% mean Average Precision at IoU threshold 0.5, representing a 6.4 percentage point improvement over the baseline model. The method maintains efficiency with 3.8M parameters and 18.6ms inference time per frame, providing a practical solution for intelligent disease detection in facility agriculture and contributing to the development of agricultural new quality productive forces.

## 1 Introduction

With the intensification of global population growth and climate change challenges, developing new quality productive forces in agriculture has become a strategic choice for ensuring food security and promoting sustainable agricultural development. New quality productive forces in agriculture emphasize the construction of efficient, green, and sustainable modern agricultural production systems through technological innovation, digital transformation, and intelligent upgrading. Against this backdrop, intelligent agricultural disease detection and recognition technology, as a core component of digital agriculture, is becoming a key technological support for driving agricultural productivity transformation.

Intelligent detection and recognition of agricultural diseases is a key technology for ensuring agricultural production and food security. With the rapid development of facility agriculture, greenhouse cultivation has become an important mode of modern agricultural production, representing a typical application of new quality productive forces in facility agriculture. Although greenhouse environments provide better disease control conditions compared to open fields, the enclosed conditions and high plant density can still facilitate rapid disease transmission when outbreaks occur, making early and accurate detection crucial for preventing significant yield losses. Statistics show that greenhouse vegetable diseases alone cause 20-30% global yield losses annually (Wójcik Gront et al., 2024). Traditional manual inspection methods are inefficient and susceptible to subjective factors in complex greenhouse environments, making it difficult to meet the monitoring needs of large-scale facility agriculture, urgently requiring revolutionary changes in detection methods through artificial intelligence technology.

Deep learning, particularly Convolutional Neural Networks (CNNs), has revolutionized computer vision with excellent feature extraction capabilities (Chowdhury et al., 2020). Classic architectures like VGG and ResNet show strong performance in disease recognition, while recent object detection advances provide new pathways for intelligent disease detection (Bonora et al., 2021; Bao et al., 2023; Mathieu et al., 2024; Jian et al., 2025). Recent advancements in Vision Transformers (ViTs), such as CrossViT (Chen et al., 2021) and DaViT (Ding et al., 2022), have demonstrated strong performance in image classification tasks. However, in greenhouse vegetable disease detection, these transformer-based architectures face significant limitations. Their computational complexity scales quadratically with input resolution, making them resource-intensive for real-time applications. While transformers excel at capturing global context, they often struggle with the fine-grained features essential for identifying small disease lesions under variable greenhouse lighting and occlusion conditions. Our proposed YOLO-vegetable model addresses these limitations through adaptive convolutional mechanisms specifically optimized for greenhouse environments.

Among various deep learning architectures, YOLO (You Only Look Once) series networks have become important models for disease detection in greenhouse environments due to their excellent real-time performance and detection accuracy. The YOLO series object detection algorithms have continuously evolved since their introduction in 2016, experiencing multiple significant upgrades from YOLOv1 to YOLOv10, achieving remarkable progress in detection accuracy, real-time performance, and resource consumption (Alif and Hussain, 2024). The recently proposed YOLOv10 further improves model detection performance in complex scenarios through optimized backbone network architecture and feature extraction strategies (Wang et al., 2024). However, existing YOLO variants face fundamental limitations in greenhouse applications due to three critical gaps: feature preservation challenges during downsampling operations, inadequate multi-scale adaptation for disease manifestations ranging from macro-level patterns to micro-level changes, and lack of dynamic feature fusion mechanisms for varying greenhouse environmental conditions.

YOLOv10 was selected as our baseline architecture for several key reasons: (1) It represents the latest advancement in the YOLO series with optimized dual-head design eliminating non-maximum suppression during inference, reducing computational overhead; (2) YOLOv10n provides the optimal balance between parameter efficiency (2.2M parameters) and detection capability, making it suitable for resource-constrained agricultural deployment scenarios; (3) Its backbone architecture incorporates modern design principles including attention mechanisms and efficient feature extraction, providing a solid foundation for our agricultural-specific modifications; (4) Extensive benchmarking shows YOLOv10 outperforms YOLOv8 and earlier versions in both accuracy and inference speed, establishing it as the current state-of-the-art for real-time object detection applications.

Vegetable disease detection in greenhouse environments faces several unique challenges. Although greenhouse environments provide more stable and controllable conditions compared to open fields, computer vision systems must still handle varying lighting conditions due to natural light changes throughout the day, reflections and scattering caused by glass or film covering materials, and shadows created by structural elements, all of which can affect image quality and detection accuracy. Dense planting leads to frequent occlusion of disease targets, increasing detection difficulty. Additionally, disease symptoms in greenhouse environments manifest in diverse forms and are often accompanied by mixed infections (Vásconez et al., 2024). These characteristics make methods that perform well in laboratory environments often struggle to achieve expected results in practical greenhouse applications. The transition from controlled laboratory settings to complex greenhouse environments highlights fundamental challenges that most existing approaches fail to address adequately.

Critical analysis of existing approaches reveals three fundamental research gaps that this work addresses: First, the feature preservation gap - most methods prioritize overall detection accuracy but fail to preserve the fine-grained visual details essential for early-stage disease detection when symptoms are subtle. Second, the scale adaptation gap - current architectures inadequately handle the multi-scale nature of disease manifestations, from macro-level patterns visible to human observers to micro-level changes detectable only through careful feature analysis. Third, the environmental adaptation gap - existing feature fusion strategies lack dynamic mechanisms to handle the varying visual complexity introduced by greenhouse environmental factors such as condensation on covering materials, structural shadows, and plant growth density variations.

However, existing research still has the following limitations: First, most methods are developed for disease images with single backgrounds under laboratory conditions, without fully considering the unique characteristics of greenhouse environments; Second, existing models perform poorly when dealing with complex situations like occlusion and lighting variations in greenhouse environments; Third, the balance between real-time performance and accuracy remains unresolved. As Bouni et al. (2024) and Abdalla et al. (2024) point out, developing detection systems adapted to complex greenhouse environments remains a challenging problem requiring urgent solutions.

To address these issues, this study proposes a vegetable disease detection method YOLO-vegetable based on improved YOLOv10 for greenhouse environments. Our experiments are validated on disease image datasets collected from multiple real greenhouse environments. The experimental data includes vegetable disease images under different lighting conditions, planting densities, and growth stages, fully reflecting the characteristics of greenhouse environments. Through comparative experiments with existing mainstream methods, we validate the superiority of our proposed method in greenhouse environments.

## 2 Literature review

Deep learning technology has made significant progress in agricultural applications, particularly demonstrating great potential in plant disease detection and recognition. Accurate recognition and early warning of vegetable diseases are crucial for ensuring agricultural production and food security. With the rapid development of computer vision and deep learning technologies, image-based automatic vegetable disease detection methods have gradually become a research hotspot (Paul et al., 2025). Deep learning methods have shown excellent performance in disease recognition tasks, mainly benefiting from their powerful feature extraction and representation capabilities. Many scholars have conducted in-depth research from different perspectives, proposing various deep learning methods based on Convolutional Neural Networks (CNNs) and object detection networks like the YOLO series (Upadhyay et al., 2025; Ali et al., 2024). Currently, research in this field mainly focuses on object detection network design, feature extraction optimization, data augmentation strategies, and multi-modal fusion.

### 2.1 Innovative strategies in object detection network design

In object detection network design, researchers have proposed multiple improvement strategies. With the development of deep learning technology, object detection networks continue to evolve. The Pruned-YOLO v5s+Shuffle model proposed by Xu et al. (2022) employs channel pruning method, achieving 93.2% detection accuracy in complex backgrounds. The Yolov5-ECA-ASFF network proposed by Zhang et al. (2024) enhances detection performance by integrating ECA and ASFF modules. Lin et al. (2024) optimized the YOLO model through combining mixed data augmentation and osprey search strategy, realizing tomato biotic stress detection. The WCG-VMamba model developed by Wang et al. (2024) introduces wavy vision Mamba network, effectively capturing semantic correlations between image features and text features, further improving detection performance in complex backgrounds. The cross-domain dynamic attention mechanism designed by Mo and Wei (2024) effectively solves uneven illumination problems. Mhala et al. (2025) addressed class imbalance issues through model compression and knowledge distillation techniques, achieving efficient model deployment. These studies indicate that object detection network design is evolving towards better adaptation to complex environments and higher accuracy.

Despite promising results in agriculture, existing YOLO-based methods still face fundamental limitations in greenhouse applications: (1) Standard strided convolutions in YOLO backbones sacrifice spatial resolution for computational efficiency, but disease symptoms often manifest as subtle texture changes requiring preservation of fine-grained details; (2) Traditional feature pyramid networks inadequately handle the extreme scale variation of disease symptoms, from macro-level leaf discoloration spanning hundreds of pixels to micro-level lesions occupying fewer than 20 pixels; (3) Fixed feature fusion weights in existing architectures cannot adapt to the dynamic visual complexity of greenhouse environments where lighting conditions, plant density, and background complexity vary significantly. Our ADEConv module specifically addresses the feature preservation challenge while maintaining computational efficiency.

### 2.2 Breakthrough progress in feature extraction optimization

In feature extraction optimization, the introduction of various innovative mechanisms has led to significant breakthroughs. Liu et al. (2021) proposed region and loss reweighting methods, providing new insights for feature extraction optimization. The EFDet model developed by Liu et al. (2024) improves detection effects in complex backgrounds by fusing features from different levels. Yan et al. (2024) proposed an adaptive deep transfer learning framework for mixed subdomains, significantly improving cross-species disease diagnosis performance. Notably, Kang et al. (2024) proposed a cascade framework combining detector and tracker, significantly reducing computational complexity while maintaining high accuracy, providing a feasible solution for practical application scenarios. Sun et al. (2025) proposed a new tomato disease recognition method based on the DeiT model, significantly improving detection accuracy in complex environments through improved feature extraction and multi-scale feature fusion mechanisms.

While attention-based approaches show promise, most existing methods apply static attention weights. Chang et al. (2024) improved wheat disease recognition through DenseNet modifications, but their approach lacks the dynamic adaptability required for greenhouse environmental variations. The AAFS mechanism differs fundamentally from existing attention approaches: Unlike SE-Net which focuses solely on channel attention through global average pooling, AAFS integrates both channel and spatial attention through parallel pathways. Compared to CBAM which applies channel and spatial attention sequentially, our approach enables simultaneous processing and dynamic weight fusion. Unlike ECA-Net’s 1D convolution for channel attention, AAFS employs adaptive group convolution with channel shuffling for enhanced feature interaction.

### 2.3 Innovative development in data augmentation strategies

In data augmentation strategies, researchers have proposed a series of innovative methods to address the unique challenges in greenhouse environments. The multi-scale feature enhancement strategy proposed by Tian et al. (2022) significantly improved the model’s recognition ability for disease regions. Karantoumanis et al. (2024) developed a strategic data augmentation method achieving a 37% accuracy improvement in legume crop disease detection. Zhang et al. (2024) proposed feature transfer and small target oversampling methods based on CycleGAN, effectively improving sample imbalance issues and successfully achieving precise recognition of early eggplant wilt disease. Johri et al. (2024) combined deep transfer learning with data augmentation, achieving significant results in small sample scenarios, providing new ideas for solving data insufficiency problems.

### 2.4 Exploration in multi-modal fusion

In multi-modal fusion, researchers have gradually begun to focus on the synergistic use of multi-source information. Yang et al. (2024) innovatively proposed a language-vision fusion framework, demonstrating excellent performance in tomato disease segmentation tasks. Hu et al. (2024) achieved deep fusion of spectral information and RGB images, significantly improving disease detection accuracy. Zhao et al. (2025) successfully implemented complementary fusion of healthy and diseased leaf information using Double Generative Adversarial Networks (DoubleGAN), providing new ideas for disease detection in small sample scenarios.

### 2.5 Small object detection challenges in complex backgrounds

Regarding small target localization and detection recognition in complex backgrounds, the unique characteristics of greenhouse environments bring distinct challenges to disease detection. Barbedo (2019) research showed that disease recognition faces challenges of small target size, blurred target features, and occlusion problems. Toda and Okura (2019) revealed the decision mechanism of CNNs in plant disease diagnosis under complex environments. Kumar et al. (2023) proposed a bidirectional feature attention pyramid network, effectively enhancing the model’s detection capability for targets of different scales. Zhou et al. (2023) innovatively introduced weakly supervised learning into disease feature segmentation, providing new approaches for small target detection. Ye et al. (2024) proposed an adaptive small target detection framework, significantly improving detection performance in low-light environments by integrating EnlightenGAN networks. Hari and Singh (2025) proposed an adaptive knowledge transfer method based on federated deep learning, significantly improving model convergence and accuracy through intelligent weight transfer technology optimizing knowledge integration between parent and child entities.

However, existing research still faces severe challenges in complex, unstructured greenhouse environments. First, image acquisition in greenhouse environments suffers from serious quality degradation issues, including image blur, noise interference, and uneven illumination, leading to significant false detections and missed detections in practical applications. Second, vegetable disease symptoms often manifest as small local areas of pathological changes, and these subtle features are easily lost during feature extraction, making them difficult to capture accurately (Qing et al., 2023). Furthermore, feature expression and multi-scale feature fusion mechanisms under complex background interference remain unresolved (Castillo-Girones et al., 2025).

To address these issues, this study proposes a high-precision localization and detection algorithm (YOLO-vegetable) for vegetable disease targets in greenhouse environments, based on the computationally efficient YOLOv10n single-stage object detection network. The algorithm contains three core innovative modules: First, we design the Adaptive Detail Enhancement Convolution (ADEConv) module, which significantly improves fine-grained feature retention capability while maintaining computational efficiency through dynamic adjustment of convolution kernel parameters; Second, we construct the Multi-granularity Feature Fusion Detection Layer (MFLayer), which achieves precise localization of small targets through hierarchical integration of feature information at different scales; Finally, we propose the Inter-layer Dynamic Fusion Pyramid Network (IDFNet), combining with Attention-guided Adaptive Feature Selection (AAFS) mechanism, significantly enhancing the model’s key information extraction capability by establishing dynamic association weights between feature layers.

## 3 Materials and methods

### 3.1 YOLO-vegetable model for vegetable disease detection

**Figure 1:**
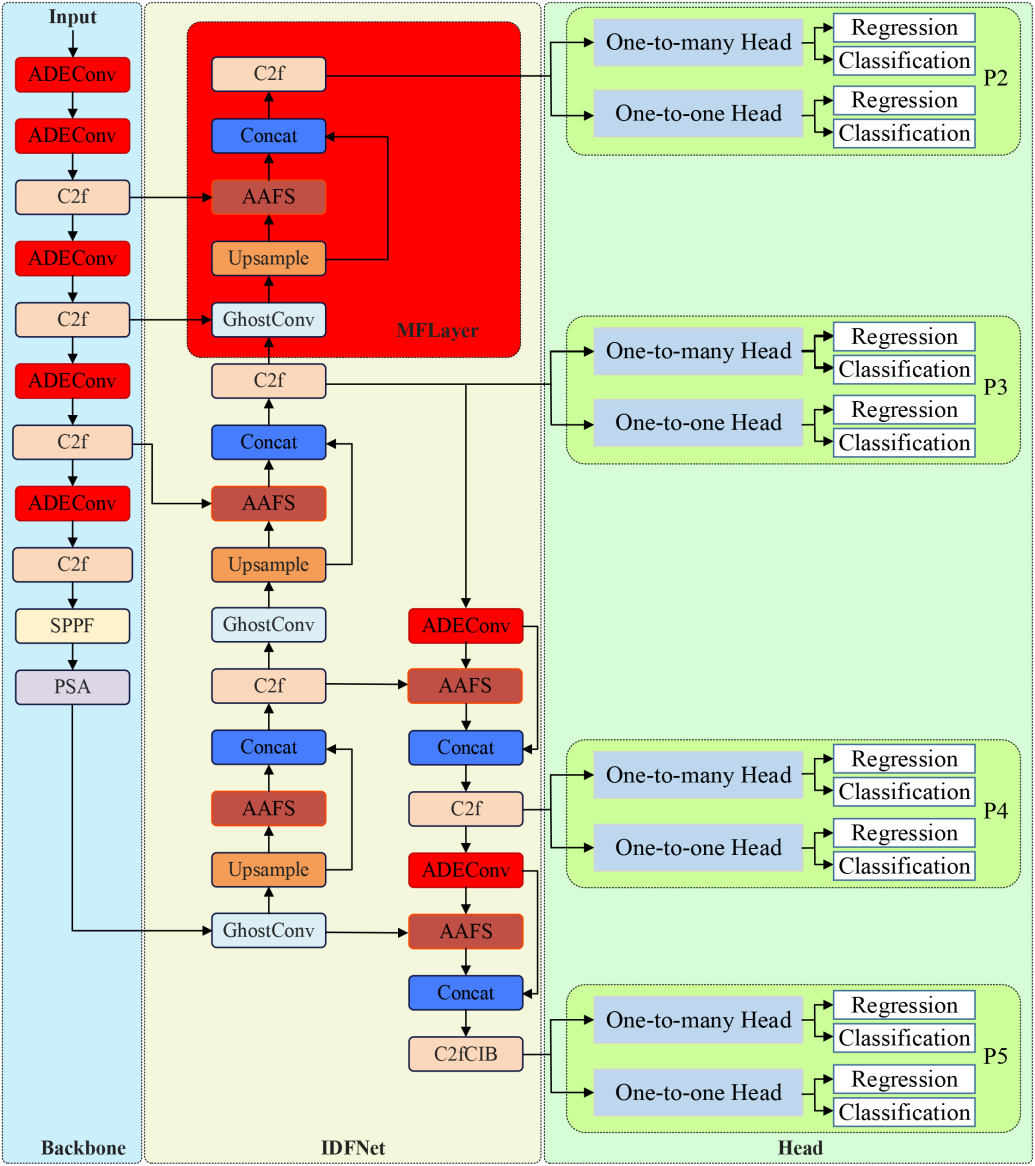
YOLO-vegetable network architecture.

Based on the characteristics of vegetable disease targets requiring detection, this study proposes a detection algorithm model YOLO-vegetable targeting greenhouse environments. Taking YOLOv10n, which has the smallest parameter count in the detection-performance-excellent YOLOv10 series, as the baseline model, we redesigned the backbone network and neck network of the original model. The structure of YOLO-vegetable is shown in **Figure 1**.

The diagram illustrates the complete network structure with backbone (left), neck network with IDFNet (center), and detection heads (right). Red boxes highlight our proposed modules: ADEConv modules replace traditional strided convolutions in the backbone, MFLayer provides multi-granularity feature fusion for small target detection, and AAFS mechanisms enable adaptive feature selection throughout the neck network. Input images (640×640) flow through the backbone for feature extraction, then through IDFNet for multi-scale feature fusion, finally reaching dual detection heads for classification and regression outputs.

#### 3.1.1 Design of ADEConv

Convolutional Neural Networks (CNNs) are widely applied in computer vision tasks. In YOLOv10 algorithm, CNN is a core part of its architecture. In traditional CNN design, strided convolution is typically used for downsampling operations to extract spatial features, with common convolution kernel sizes of 3×3 or larger. Strided convolution achieves downsampling by setting stride greater than 1 during convolution operations, meaning the convolution kernel moves multiple pixels at a time rather than pixel by pixel. For example, when stride is set to 2, the convolution kernel moves 2 pixels each time, where only one out of every two pixels in the input feature map is covered by the convolution kernel, thus halving the output feature map dimensions. Because strided convolution skips some input data, important local information may not be captured. Although this feature downsampling can aggregate contextual information and achieve dimension reduction, it comes at the cost of losing detail information, challenging the model’s ability to recognize and learn small target features.

Traditional pooling layers can also reduce feature map resolution and computational cost, but during this process, information about small objects may be excessively compressed or completely lost, leading to decreased detection performance. Therefore, when using these modules for downsampling, details of vegetable disease targets are inevitably lost, affecting the network’s ability to extract fine details of small disease spots. Moreover, diseased areas in vegetable images occupy extremely small areas, and uneven lighting in greenhouse environments makes it necessary for the network to extract more detailed information to improve small target recognition ability.

**Figure 2:**
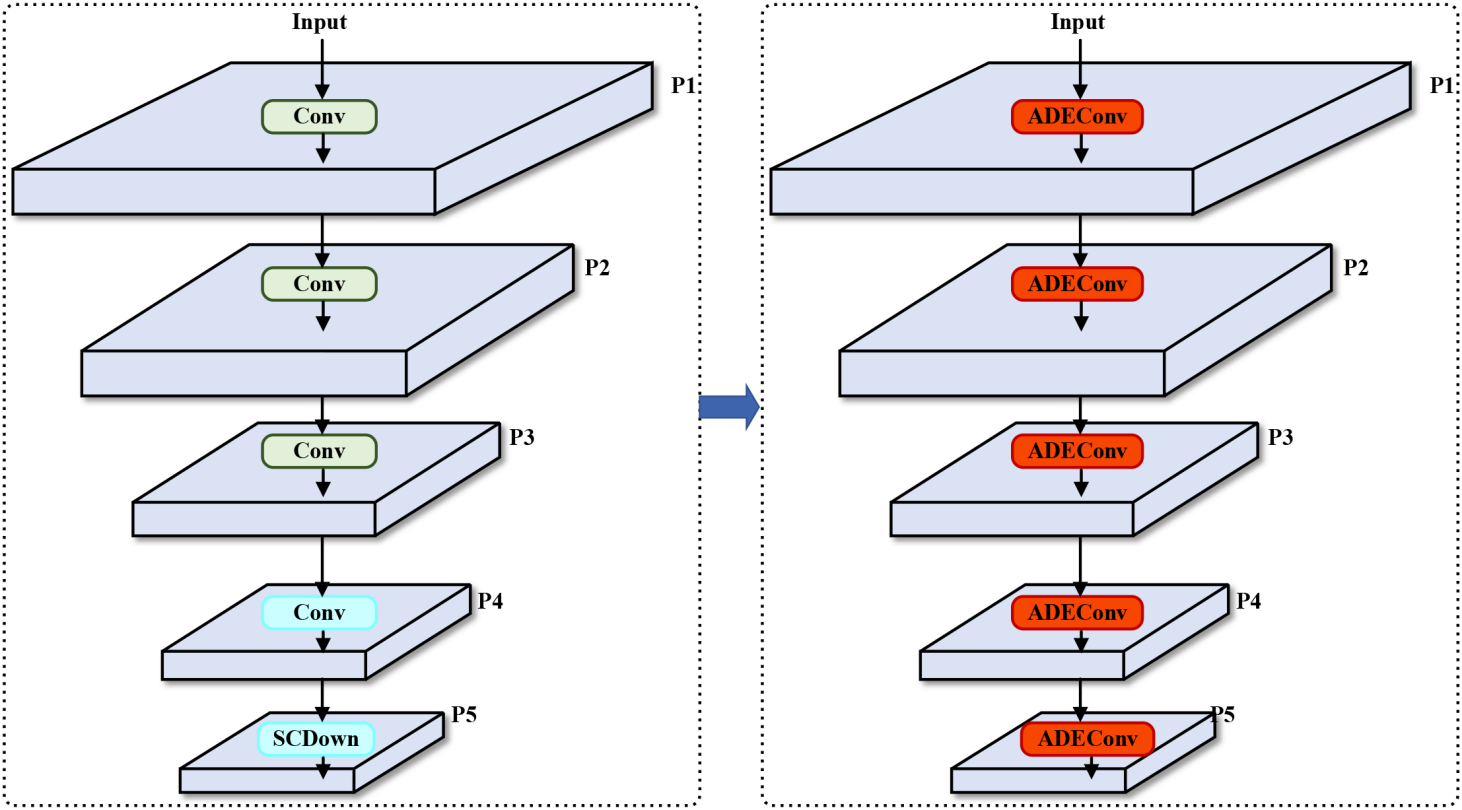
Backbone network architecture comparison. Left: Original YOLOv10n backbone using standard strided convolutions (Conv) and SCDown modules. Right: Our improved backbone with ADEConv modules replacing all downsampling operations. The ADEConv modules preserve fine-grained features while achieving the same spatial dimension reduction, addressing the information loss problem inherent in traditional strided convolutions. Each P1-P5 represents feature maps at different scales (1/2, 1/4, 1/8, 1/16, 1/32 of input resolution respectively).

To address the aforementioned issues, this study replaces the traditional strided convolution modules in YOLOv10’s backbone network with ADEConv modules, which improve small object detection performance by preserving fine-grained information and avoiding excessive compression of image features. The replacement process is shown in **Figure 2**.

**Figure 3:**
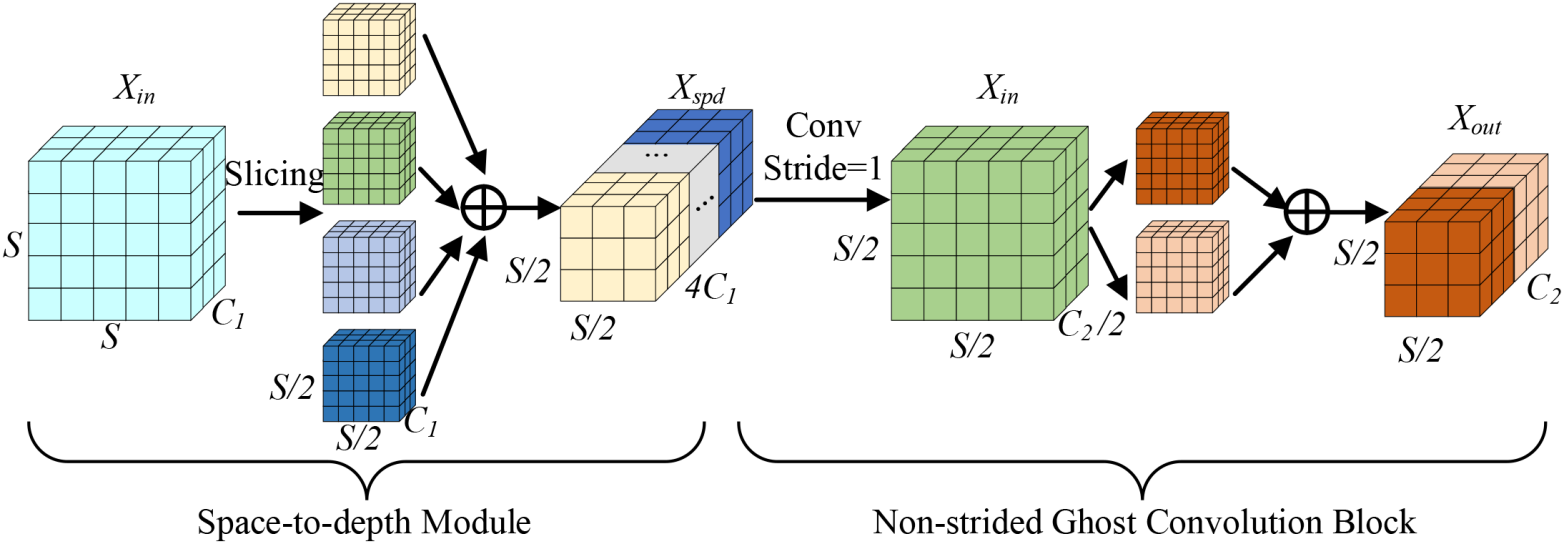
ADEConv module architecture.

The ADEConv module primarily consists of a Space-to-depth Module and a Non-strided Ghost Convolution Block (Han et al., 2020), replacing all strided convolution blocks in YOLOv10’s backbone network. The Space-to-depth Module first performs pixel-wise division and rearranges pixels from each block into depth channels, achieving spatial compression of the input feature map. This reorganization not only halves the feature map’s spatial dimensions but also preserves all original information of the processed pixels, effectively avoiding potential detail loss that might occur during traditional strided convolution’s spatial compression process. The module’s main structure is shown in **Figure 3**.

Here, $X_{in}$ denotes the ADEConv module’s input feature map, S represents the spatial dimension width/height value of the input feature map, $C_{1}$ is the input feature map’s channel number, $X_{spd}$ is the Space-to-depth Module’s output feature map, $C_{2}$ is the output feature map’s channel number, and $X_{out}$ is the ADEConv module’s output feature map.

The first operation of the Space-to-depth Module is feature map slicing, with its formula being:


(1) $$f_{h,w}=X\left[h:S:scale,w:S:scale\right]$$

where X denotes the input feature map, h and w are the starting indices for feature map height and width respectively, S is the input feature map dimension, and scale is the slicing stride. When scale=2, extracting values every 2 elements yields the following four feature maps:


(2) $$\{\begin{array}{c} f_{0,0}=X\left[0:S:2,0:S:2\right]\\ f_{0,1}=X\left[0:S:2,1:S:2\right]\\ f_{1,0}=X\left[1:S:2,0:S:2\right]\\ f_{1,1}=X\left[1:S:2,1:S:2\right] \end{array}$$

Finally, channel concatenation is performed:


(3) $$X_{spd}=Concat\left[f_{0,0}:f_{0,1}:f_{1,0}:f_{1,1}\right]$$

where Concat[] represents the Channel-wise Concatenation operation. While preserving detail information, the Space-to-depth Module reduces the feature map’s spatial dimensions. Subsequently, the Non-strided Ghost Convolution Block reduces channel numbers, with its formula being:


(4) $$X_{out}=Concat\left[GC_{5\times 5}\left(F_{C_{2}/2}\left(X_{spd}\right)\right):F_{C_{2}/2}\left(X_{spd}\right)\right]$$

where $GC_{5\times 5}$ represents group convolution operation with a 5×5 kernel size, $F_{C_{2}/2}$ represents a 1×1 convolution transformation function using channel size of $C_{2}/2$ . Through these operations, the ADEConv module can achieve downsampling operations while maximally preserving all detail information from the original image without significantly increasing computational cost.

#### 3.1.2 Design of MFLayer

In traditional YOLO series network design, the Path Aggregation Feature Pyramid Network (PAFPN) adopts a structure of downsampling followed by upsampling then downsampling again, combined with skip connections to enhance information exchange between feature maps. Feature maps are divided into five levels from P1 to P5 based on their spatial reduction ratio relative to the input image (1/2, 1/4, 1/8, 1/16, 1/32).

After multiple downsampling operations, some low-level features may gradually be lost. Although skip connections between feature maps of the same level during downsampling and upsampling help recover detail information lost due to consecutive convolutions and pooling operations, for extremely small targets, the original structure’s restoration of details remains insufficient after five downsampling operations followed by only two upsampling operations, affecting network detection performance.

**Figure 4:**
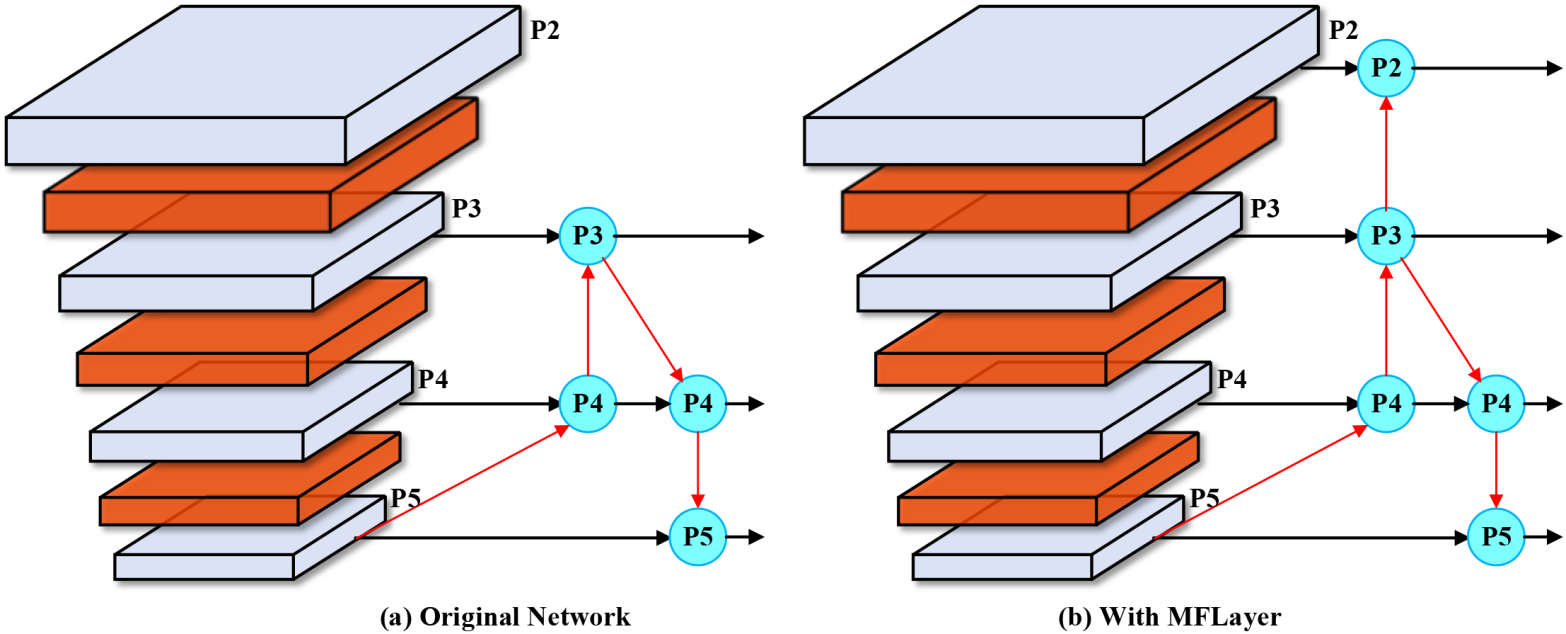
MFLayer schematic diagram. **(a)** Original Network: Traditional YOLO architecture processes features through standard downsampling and upsampling paths, with P2-P5 representing feature pyramid levels at different scales. **(b)** With MFLayer: Our enhanced architecture introduces additional connections (red arrows) that preserve high-resolution P2 features and directly integrate them with upsampled deep features, enabling better small target detection through multi-granularity feature fusion.

To address this issue, we introduce the MFLayer in the neck network to preserve extremely small target detail features. We fuse the P2 feature layer with downsampling factor of two from the backbone network and the P2 feature layer obtained after three upsampling operations from the P5 feature layer, and directly use it as input for the small target detection head. This design aims to enhance the model’s localization and recognition capability for extremely small-sized objects by preserving sufficient low-level features. The principle of the MFLayer is shown in **Figure 4**.

As shown in **Figure 4**, (a) Original Network: Traditional YOLO architecture processes features through standard downsampling and upsampling paths, with P2-P5 representing feature pyramid levels. (b) With MFLayer: Our enhanced architecture introduces additional connections (red arrows) that preserve high-resolution P2 features and directly integrate them with upsampled deep features. This strategy of combining low-level detail features with high-level semantic features not only helps improve detection effects for small targets but can also maintain the model’s computational efficiency to some extent. Through this approach, the model can more accurately capture and identify small objects in images. The MFLayer design offers significant advantages over traditional feature fusion approaches by establishing direct connections between high-resolution and low-resolution feature maps. This capability directly addresses one of the most significant challenges in greenhouse disease detection, where early-stage symptoms often manifest as subtle lesions easily lost during conventional feature downsampling.

#### 3.1.3 Design of IDFNet

Traditional YOLO series networks use PAFPN as their neck network structure, where there is no information exchange between each feature map layer and the backbone, potentially leading to loss of some detail features. The preservation of detail features is crucial for small target recognition. The Bi-directional Feature Pyramid Network (BiFPN) adds cross-scale fusion layers compared to PAFPN (Tan et al., 2020), achieving feature flow from top-down and bottom-up, and optimizing the feature fusion process by adding weights to each feature input, which helps preserve more useful information. Since BiFPN introduces dynamic weights, these weights are optimized through backpropagation during network training, which might increase the network’s computational burden and potentially lead to training instability in early stages due to uncertainty in initial weight values.

**Figure 5:**
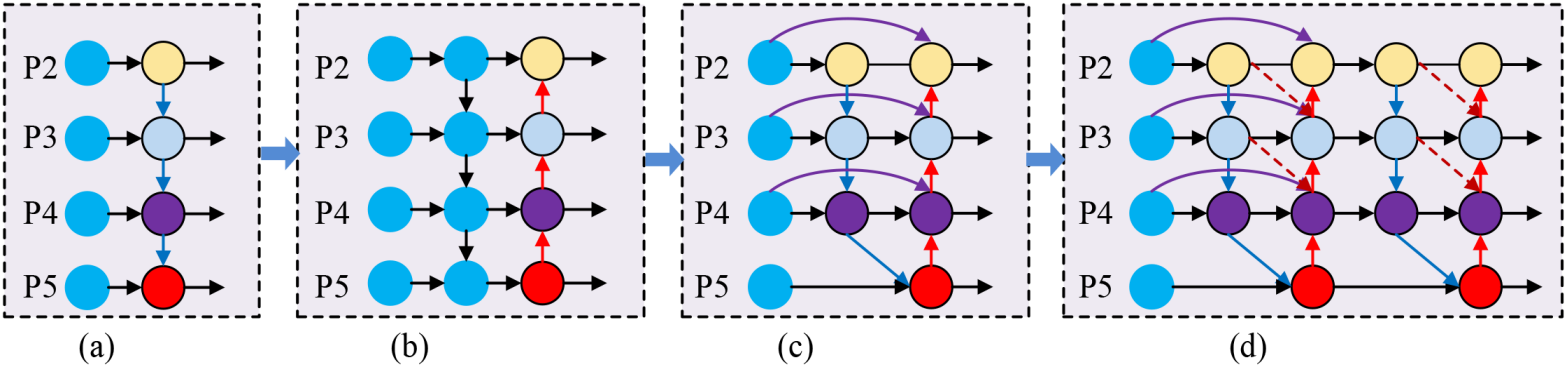
Comparison of feature pyramid network architectures. **(a)** FPN: Basic top-down feature fusion with unidirectional information flow from high-level to low-level features. **(b)** PAFPN: Bidirectional feature fusion with additional bottom-up pathway enabling information exchange between different pyramid levels. **(c)** BiFPN: Weighted bidirectional fusion with cross-scale connections and learnable fusion weights. **(d)** IDFNet (Ours): Enhanced architecture with additional backbone-to-neck connections (green arrows) and dynamic fusion weights through AAFS mechanism for improved feature propagation.

To address these issues, this study redesigns the original neck network, proposing the IDFNet. This network introduces a feature propagation path from backbone to downsampling path to reduce the loss of small target features during propagation. By introducing cross-layer feature propagation paths, we establish direct connections between the backbone network and feature pyramid network, significantly reducing information loss of fine-grained features during multiple downsampling processes. The output layers from the feature extraction network are fed into P3 layer (low-level features), P4 layer (mid-level features), and P5 layer (high-level features), and BiFPN fusion method is repeated 3 times between P3, P4, and P5 layers, implementing multi-scale feature fusion. Each fusion can extract higher-level, more abstract features based on existing foundations, improving detection accuracy. The overall architecture of IDFNet is shown in **Figure 5**.

As shown in **Figure 5**, (a) FPN: Basic top-down feature fusion. (b) PAFPN: Bidirectional feature fusion with additional bottom-up pathway. (c) BiFPN: Weighted bidirectional fusion with cross-scale connections. (d) IDFNet (Ours): Enhanced architecture with additional backbone-to-neck connections (green arrows) and dynamic fusion weights.

**Figure 6:**
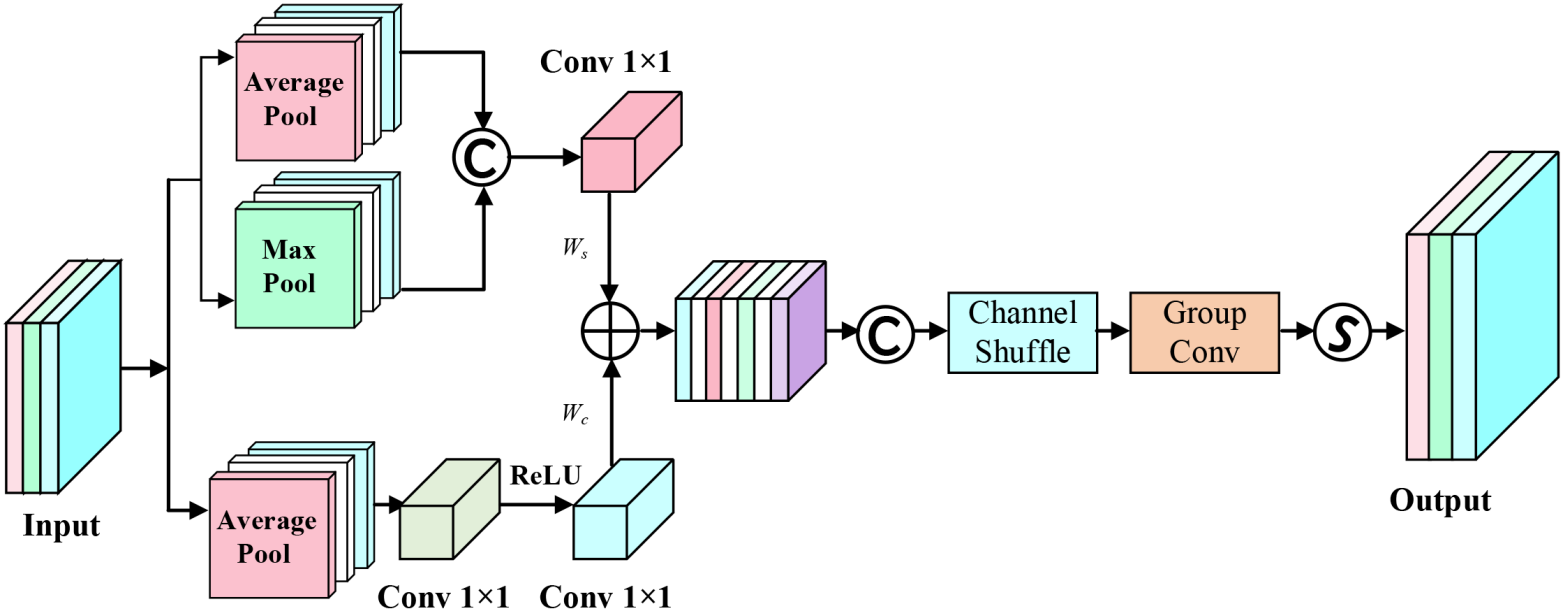
AAFS module architecture.

Simultaneously, we design the AAFS mechanism as the core feature fusion strategy. Unlike traditional BiFPN using fixed weight allocation methods, the AAFS mechanism dynamically calculates fusion weights by comprehensively analyzing channel-dimension and spatial-dimension correlations of feature maps, enabling the network to adaptively enhance features crucial for detection tasks. This strategy based on feature correlation adaptive selection not only improves the model’s detection sensitivity to subtle disease features but also enhances feature expression’s discriminative ability across different scales. The principle of AAFS is shown in **Figure 6**.

Let X be the feature map input, obtaining its channel-dimension and spatial-dimension features:


(5) Wc=FC×1×1(max(0,FCr×1×1(XGAPC) ))

(6) $$W_{s}=F_{C\times 7\times 7}\left(\left[X_{GAP}^{s},X_{GMP}^{s}\right]\right)$$

where $W_{c}$ represents channel features, $W_{s}$ represents spatial features, $F_{C\times 1\times 1}$ represents 1×1 convolution transformation function with C channels, r is the channel reduction ratio, max(0,·) represents the ReLU activation function, $X_{GAP}^{C}$ represents global average pooling operation across spatial dimensions, $X_{GAP}^{s}$ and $X_{GMP}^{s}$ represent global average pooling and global max pooling operations across channel dimensions respectively.

Subsequently, the features from both dimensions are added and concatenated with input X, followed by channel shuffling operation, then passing through group convolution and Sigmoid operation to obtain fusion weight W:


(7) $$W=\sigma \left(GC_{7\times 7}CS\left(\left(Concat\left[X,W_{c}+W_{s}\right]\right)\right)\right)$$

where σ represents Sigmoid operation, $GC_{7\times 7}$ represents group convolution operation with 5×5 kernel size, CS() represents channel shuffle operation.

#### 3.1.4 Variable definitions

**Table 1** summarizes key variables, their symbols, definitions, and numerical values/ranges used in the study.

**Table 1:** Variable definitions.

Symbol	Definition	Value/Range	Source
*C*1​	Input channels of ADEConv	*C*1​=64	Backbone network configuration
*C*2​	Output channels of ADEConv	*C*2​=128	Equation 4
*H*,*W*	Height/Width of input feature maps	*H*=*W*=640	Image resolution setting
*σ*	Gaussian noise intensity	σ∈[0.1,0.3] *σ*∈[0.1,0.3]	Noise robustness experiments
*α*	Learning rate decay factor	*α*=0.95	Training hyperparameters
*r*	Channel reduction ratio in AAFS	*r*=4	Equation 5

### 3.2 Vegetable disease image dataset

**Figure 7:**
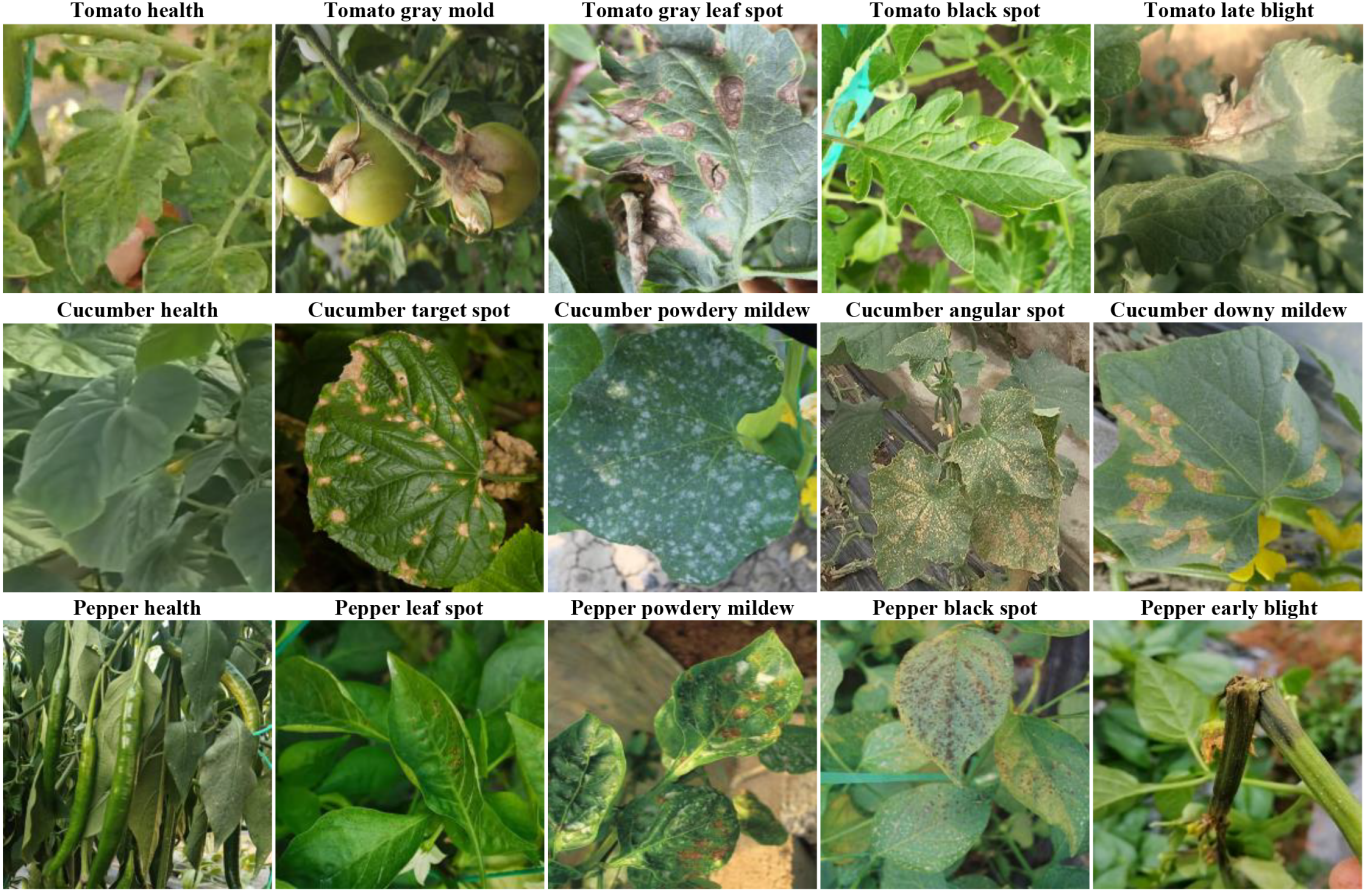
Selected samples of vegetable disease images.

To ensure the dataset encompasses diverse greenhouse environments and meets the model’s requirements for handling complex backgrounds, occlusion, blurred disease features, and small target detection, this study employs our self-built Vegetable Disease Dataset (VDD), comprising 15,000 images involving 3 major facility vegetables (tomato, cucumber, pepper) and their 15 common diseases along with healthy samples (**Figure 7**; **Table 2**). Data collection was conducted in controlled greenhouse facilities with temperature at 22-28°C (day) and 18-22°C (night), and relative humidity at 60-75%. Images were captured using professional high-resolution cameras at 30-50cm distance across four growth stages (seedling, vegetative, flowering, fruiting) under diverse weather conditions to ensure dataset robustness. The dataset is annotated following YOLO format specifications. Dataset annotation was performed by certified plant pathologists following standardized protocols. Each disease instance was annotated with precise bounding boxes. The dataset is divided into training set, validation set, and test set in a 7:2:1 ratio. This dataset contains vegetable disease targets under various weather conditions.

**Table 2:** Sample counts of vegetable disease types.

No.	Disease type	Number of images		
		Training set	Validation set	Test set
A1	Tomato health	700	200	100
A2	Tomato gray mold	700	200	100
A3	Tomato gray leaf spot	700	200	100
A4	Tomato black spot	700	200	100
A5	Tomato late blight	700	200	100
B1	Cucumber health	700	200	100
B2	Cucumber target spot	700	200	100
B3	Cucumber powdery mildew	700	200	100
B4	Cucumber angular spot	700	200	100
B5	Cucumber downy mildew	700	200	100
C1	Pepper health	700	200	100
C2	Pepper leaf spot	700	200	100
C3	Pepper powdery mildew	700	200	100
C4	Pepper black spot	700	200	100
C5	Pepper early blight	700	200	100
Total		10500	3000	1500

**Figure 7** showcases representative samples from our dataset, illustrating the diversity of disease manifestations across different vegetable types and growth stages. The images demonstrate varying symptom presentations, from early-stage subtle discolorations to advanced necrotic lesions, captured under diverse lighting conditions and viewing angles. This diversity ensures models trained on our dataset develop robust generalization capabilities applicable to real-world greenhouse scenarios.

## 4 Results and discussion

### 4.1 Experimental environment and parameter configuration

The experimental platform uses Ubuntu22.04 as the operating system, equipped with Intel(R) Xeon(R) Gold 5418Y processor with a main frequency of 2.00 GHz. The system memory is 32GB, with an Nvidia GeForce RTX 4090 graphics card having 24GB memory capacity. The PyTorch framework version is 2.2.2+cu121, and Python version is 3.10.0. Input image resolution is uniformly set to 640×640 to ensure the clarity of targets at different scales in feature maps, adapting to the model’s requirements for small target detection. During training, the model’s initial learning rate is set to 0.01, batch size to 16, momentum to 0.937, weight decay coefficient to 0.0005, and training epochs to 100. To further enhance the model’s robustness, all experiments are conducted without any form of pre-trained weights, and all experiments use consistent hyperparameters for training and validation to ensure comparability of experimental results.

Data augmentation techniques, including mosaic and mixup, were applied to enhance dataset diversity. Our augmentation pipeline also included random rotation (± 15°), horizontal and vertical flipping, and adjustments to brightness (± 25%), contrast (± 20%), and saturation (± 15%) to simulate the variable lighting conditions in greenhouse environments. To address class imbalance issues, we employed oversampling for minority disease classes, ensuring balanced representation during training while maintaining authentic image characteristics.

For hyperparameter optimization, we conducted a systematic grid search to identify optimal values. The learning rate was initialized at 0.01 and adjusted using a cosine annealing scheduler with warm restarts. Weight decay was set to 0.0005, and momentum maintained at 0.937 throughout training. These parameters were selected after evaluating 16 different configurations, with the final values providing the best balance between convergence speed and model generalization.

### 4.2 Evaluation metrics

To comprehensively evaluate YOLO-vegetable model’s balanced performance in terms of speed and accuracy, this study selects Precision, Recall, Average Precision (AP), Mean Average Precision (mAP), Parameters, FLOPs, and Inference Time as evaluation metrics. In object detection tasks, mAP@0.5 and mAP@0.5:0.95 serve as primary evaluation metrics, capable of comprehensively evaluating model performance. Specifically, mAP@0.5 represents average precision at Intersection over Union (IoU) threshold of 0.5; mAP@0.5:0.95 reflects model stability under different IoU thresholds. Meanwhile, through evaluating parameter count and computational complexity, we provide important references for practical deployment.

### 4.3 Experimental process

**Figure 8:**
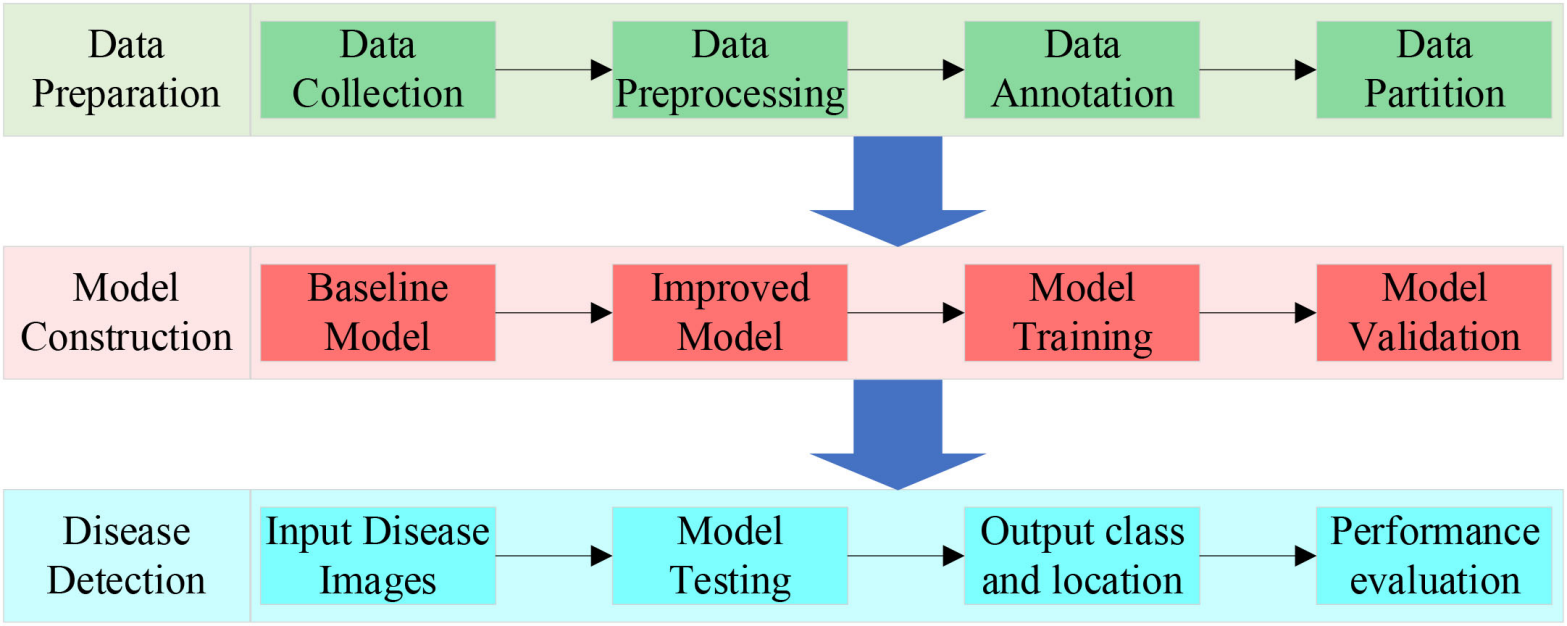
The flow chart of vegetable disease detection.

To significantly improve the accuracy and efficiency of vegetable disease target detection, we propose YOLO-vegetable. The experimental process includes three key phases, as shown in **Figure 8**.

**Figure 9:**
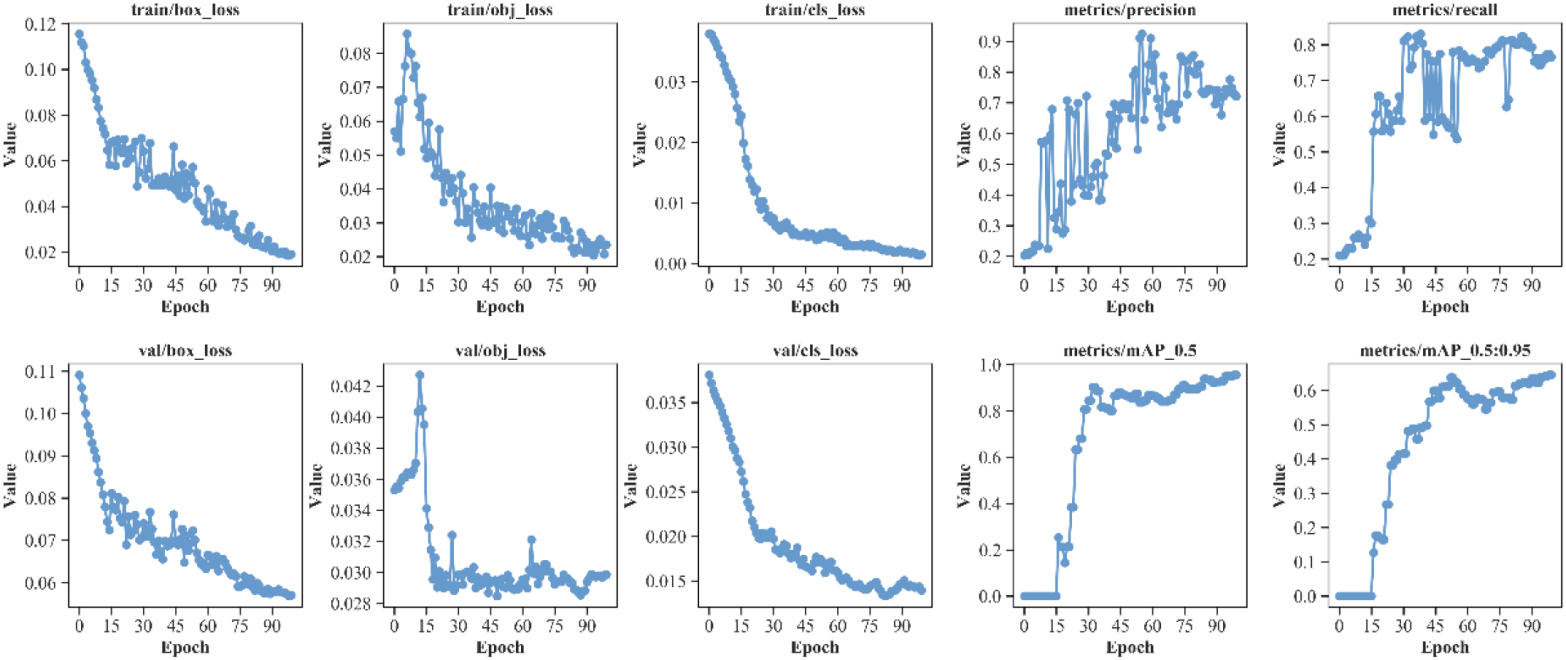
Performance metrics of the proposed YOLO-vegetable model.

To validate the effectiveness of the proposed model, experiments were conducted on our self-built vegetable disease dataset. The training and validation curves of the proposed model’s box loss, dfl loss, classification loss, and other performance metrics including precision, recall, mAP@0.5, and mAP@0.5:0.95 are shown in **Figure 9**, with iteration count on the horizontal axis.

As shown in **Figure 9**, during the 100 training iterations, the loss exhibit stable convergence patterns, gradually stabilizing as training progresses. Similarly, the validation loss demonstrate consistent convergence behavior, reaching steady states by the final epochs. Observing the model’s mAP@0.5 and mAP@0.5:0.95 convergence curves, performance metric curves begin to stabilize after 50 iterations. Finally, the model achieves excellent performance on the test set: mAP@0.5 reaches around 95%, and mAP@0.5:0.95 reaches approximately 60%. Meanwhile, the model demonstrates good precision and recall performance, indicating strong generalization ability and stability in vegetable detection tasks. The YOLO-vegetable model achieves a parameter count of 3.8M and a computational complexity of 14.7 GFLOPs, making it highly efficient for real-time deployment in resource-constrained environments.

### 4.4 Experimental results

To comprehensively evaluate YOLO-vegetable model’s performance in detecting various vegetable diseases, testing was conducted based on our self-built dataset. **Table 3** shows the detection results of YOLO-vegetable model for these different types of diseases.

**Table 3:** Detection results for different disease types.

No.	Disease type	Precision(%)	Recall(%)	AP50(%)
A1	Tomato health	96.8	95.4	96.2
A2	Tomato gray mold	95.2	94.8	95.0
A3	Tomato gray leaf spot	94.6	93.9	94.3
A4	Tomato black spot	95.8	94.7	95.3
A5	Tomato late blight	96.2	95.1	95.7
B1	Cucumber health	97.1	96.3	96.8
B2	Cucumber target spot	95.4	94.6	95.1
B3	Cucumber powdery mildew	94.8	93.9	94.4
B4	Cucumber angular spot	95.6	94.8	95.2
B5	Cucumber downy mildew	96.4	95.2	95.9
C1	Pepper health	97.3	96.5	97.0
C2	Pepper leaf spot	95.7	94.9	95.3
C3	Pepper powdery mildew	94.9	94.2	94.6
C4	Pepper black spot	95.8	94.7	95.3
C5	Pepper early blight	96.1	95.3	95.8
	Average	95.8	94.9	95.6

As shown in **Table 3**, YOLO-vegetable model achieves Precision, Recall, and AP values above 90% for 15 vegetable diseases and healthy samples, demonstrating high precision and recall rates. The model’s mAP reaches 95.6%, fully proving its excellent performance in handling different types of vegetable diseases. Additionally, the model’s outstanding performance in healthy sample recognition helps reduce misdiagnosis and unnecessary treatments.

**Figure 10:**
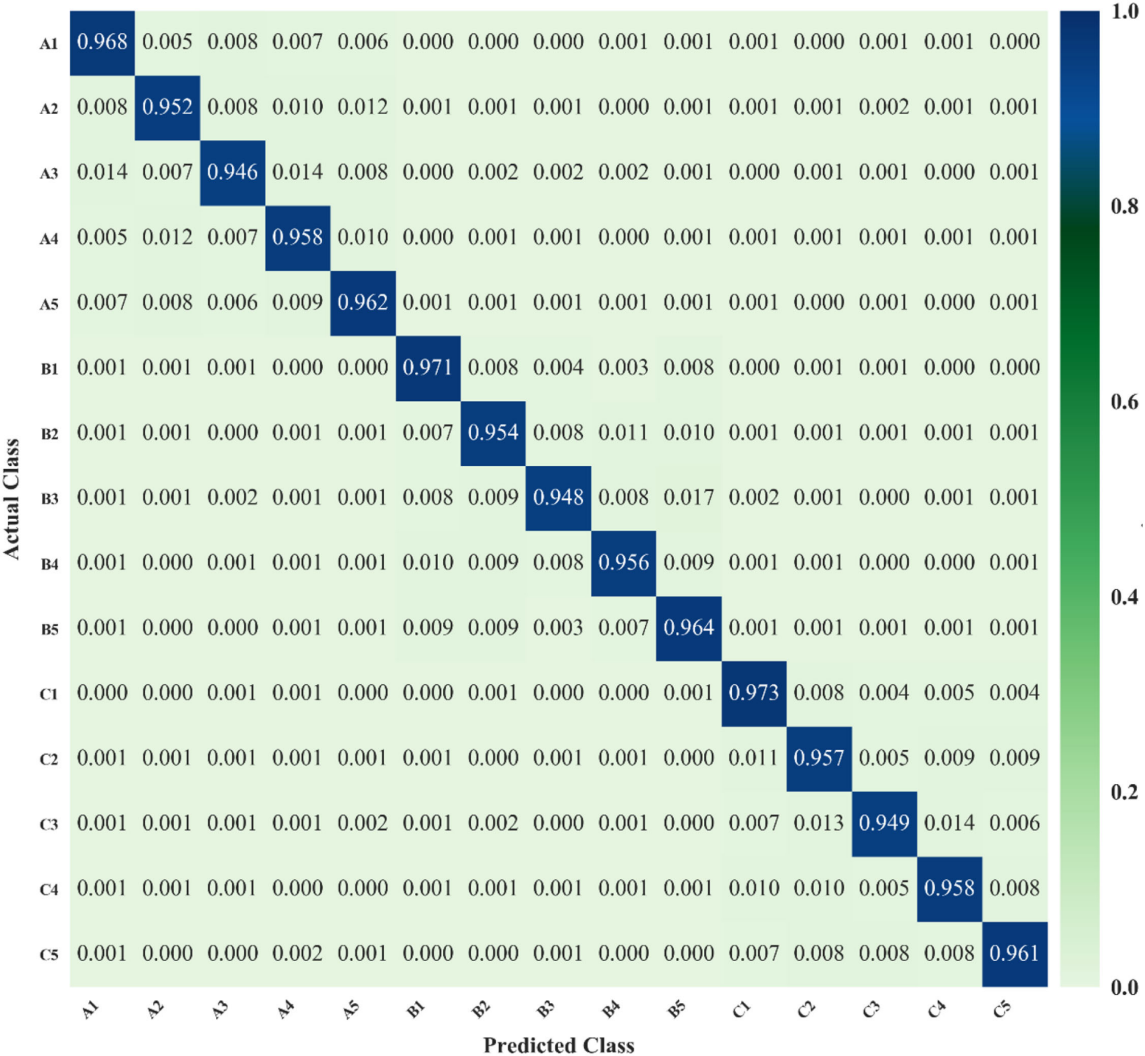
Confusion matrix of the proposed YOLO-vegetable model.

**Figure 10** presents the confusion matrix of our proposed YOLO-vegetable model, showing the proportion of detection results for each category. The horizontal axis represents predicted class numbers, while the vertical axis represents annotated class numbers. In the matrix, squares where predicted classes match annotated classes represent correct algorithm predictions, while other squares represent class confusion cases. From the prediction results, the model demonstrates high detection accuracy with minimal confusion overall, showing only a small proportion of class confusion cases.

**Figure 11:**
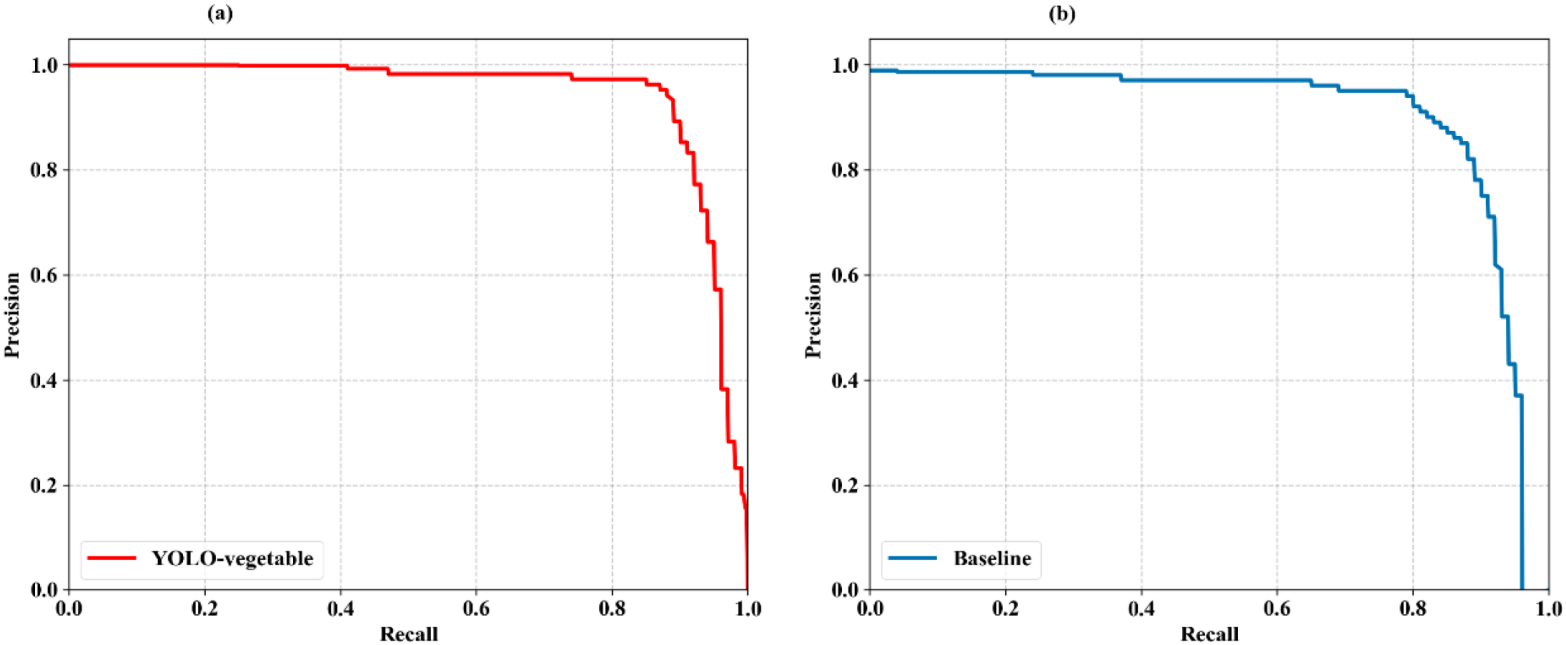
PR curves of the proposed YOLO-vegetable model compared to the baseline model. **(a)** YOLO-vegetable: Precision-Recall curve showing superior performance with AP of 0.956, maintaining higher precision values across a wider range of recall thresholds. **(b)** Baseline: YOLOv10n baseline model PR curve with AP of 0.892, demonstrating lower overall detection performance compared to our proposed method.

To comprehensively evaluate the detection performance of the proposed YOLO-vegetable model compared to the baseline model, we analyzed the Precision-Recall (PR) curves, which illustrate the trade-off between precision and recall at different confidence thresholds. **Figure 11** presents the PR curves for both models.

The PR curve analysis reveals that the proposed YOLO-vegetable model (**Figure 11a**) achieves superior performance with an Average Precision (AP) of 0.956, representing a significant improvement over the baseline model’s AP of 0.892 (**Figure 11b**). The YOLO-vegetable model maintains higher precision values across a wider range of recall values, indicating its ability to identify disease instances correctly while minimizing false positives. The enhanced performance demonstrated by the PR curves further validates the effectiveness of our architectural improvements—specifically the ADEConv module for preserving fine-grained features, the MFLayer for accurate small target localization, and the IDFNet for enhanced feature fusion. These components collectively contribute to the model’s ability to maintain high precision even at high recall thresholds, making it well-suited for real-world greenhouse disease detection scenarios with varying lighting conditions and complex backgrounds.

### 4.5 Ablation study

To systematically evaluate the performance contribution of each core module in the YOLO-vegetable algorithm, this study uses YOLOv10n as the baseline model, progressively introducing the ADEConv, MFLayer, and IDFNet modules. Through comprehensive analysis of model accuracy, computational complexity, and inference time, we validate the optimization effect of each module. Detailed experimental results are shown in **Table 4**.

**Table 4:** Ablation study results.

Group	ADEConv	MFLayer	IDFNet	mAP (%)	Parameters (M)	FLOPs (G)	Time (ms)
1	No	No	No	89.2	2.2	6.5	15.6
2	Yes	No	No	94.3	3.6	9.5	16.1
3	No	Yes	No	93.2	2.8	15.1	18.2
4	No	No	Yes	94.3	2.7	8.3	15.9
5	Yes	Yes	No	94.5	3.8	16.7	20.1
6	Yes	No	Yes	94.2	4.2	10.3	17.6
7	No	Yes	Yes	94.0	3.4	12.9	17.9
8	Yes	Yes	Yes	95.6	3.8	14.7	18.6

The experimental results show that introducing the ADEConv module improves mAP@0.5 from 89.2% to 94.3%, significantly enhancing the network’s fine-grained feature extraction capability. Although parameter count increases from 2.2M to 3.6M and computational cost increases to 9.5 GFLOPs, it only brings a 0.5ms inference time delay (15.6ms to 16.1ms). While the MFLayer module leads to computational cost increasing to 15.1 GFLOPs with a 2.6ms inference time increase, it performs excellently in maintaining small target detail features, achieving 93.2% mAP@0.5 with only 2.8M parameters. The introduction of IDFNet demonstrates superior feature fusion effects, achieving 94.3% mAP@0.5 with just 2.7M parameters, while maintaining comparable computational cost (8.3 GFLOPs) and inference time (15.9ms).

Further research reveals that the combination of ADEConv and MFLayer achieves 94.5% mAP@0.5, surpassing single-module applications. Although computational cost increases to 16.7 GFLOPs, through reasonable parameter configuration (3.8M), the inference time increase (20.1ms) remains acceptable. This result demonstrates the synergistic effect between detail feature extraction and feature preservation. Building upon this foundation, introducing IDFNet to form the complete YOLO-vegetable model not only further improves mAP@0.5 to 95.6% but also achieves optimization in computational resource utilization: maintaining parameter count at 3.8M, reducing computational cost to 14.7 GFLOPs, and controlling inference time to 18.6ms. This balance between performance improvement and computational overhead fully validates the necessity of innovative modules and their excellent synergistic effects.

**Figure 12:**
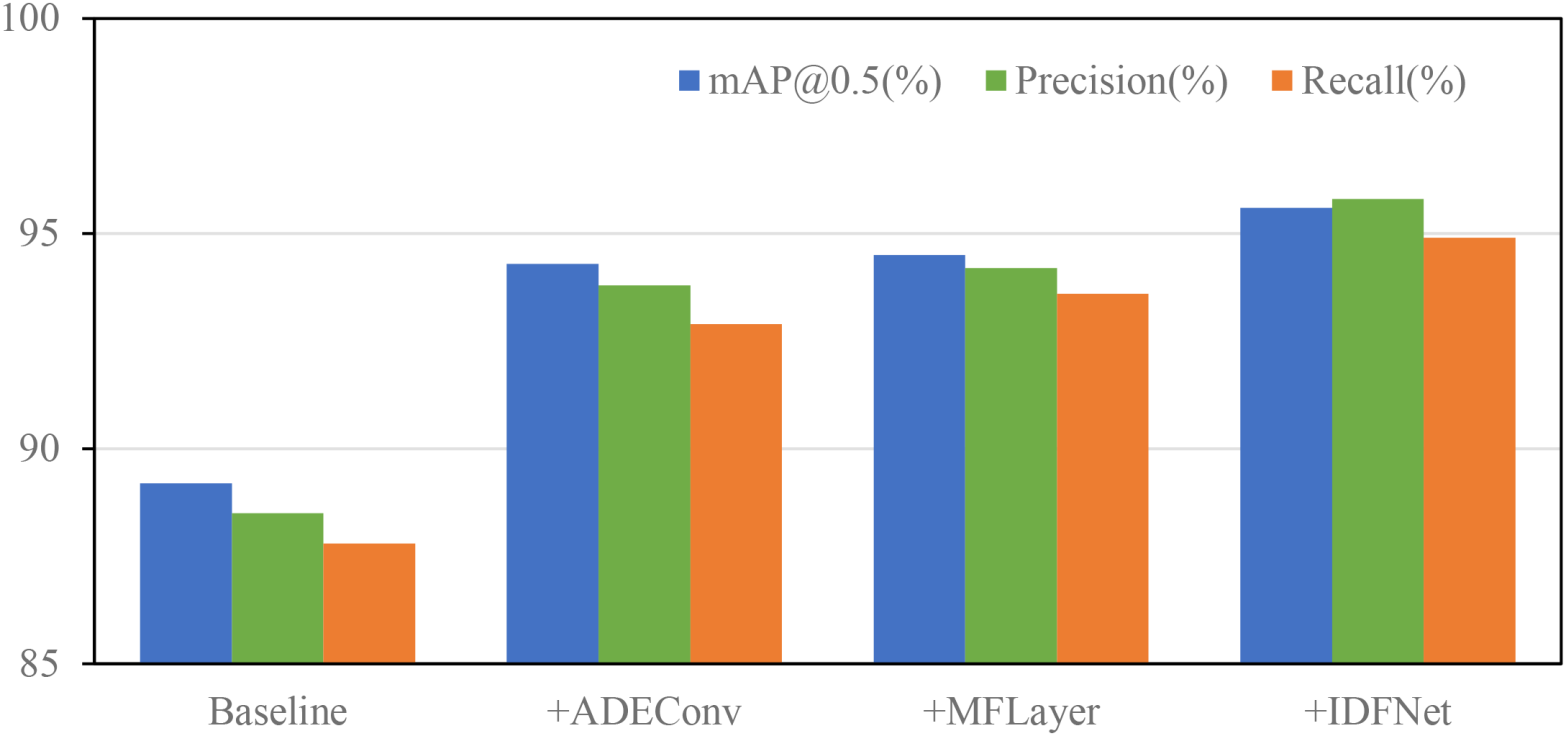
Performance contribution comparison of different modules.

To more intuitively demonstrate the performance improvement effects of different modules on the model, **Figure 12** illustrates the trends of model performance as different modules are introduced. From the overall trends in **Figure 12**, the progressive introduction of the three innovative modules shows steady performance improvement, with balanced enhancement across all metrics, demonstrating no significant degradation in any indicator while others improve. This balanced performance improvement validates that our proposed improvement strategies are not only necessary but can work collaboratively and mutually reinforce each other, achieving overall optimization of model performance.

**Figure 13:**
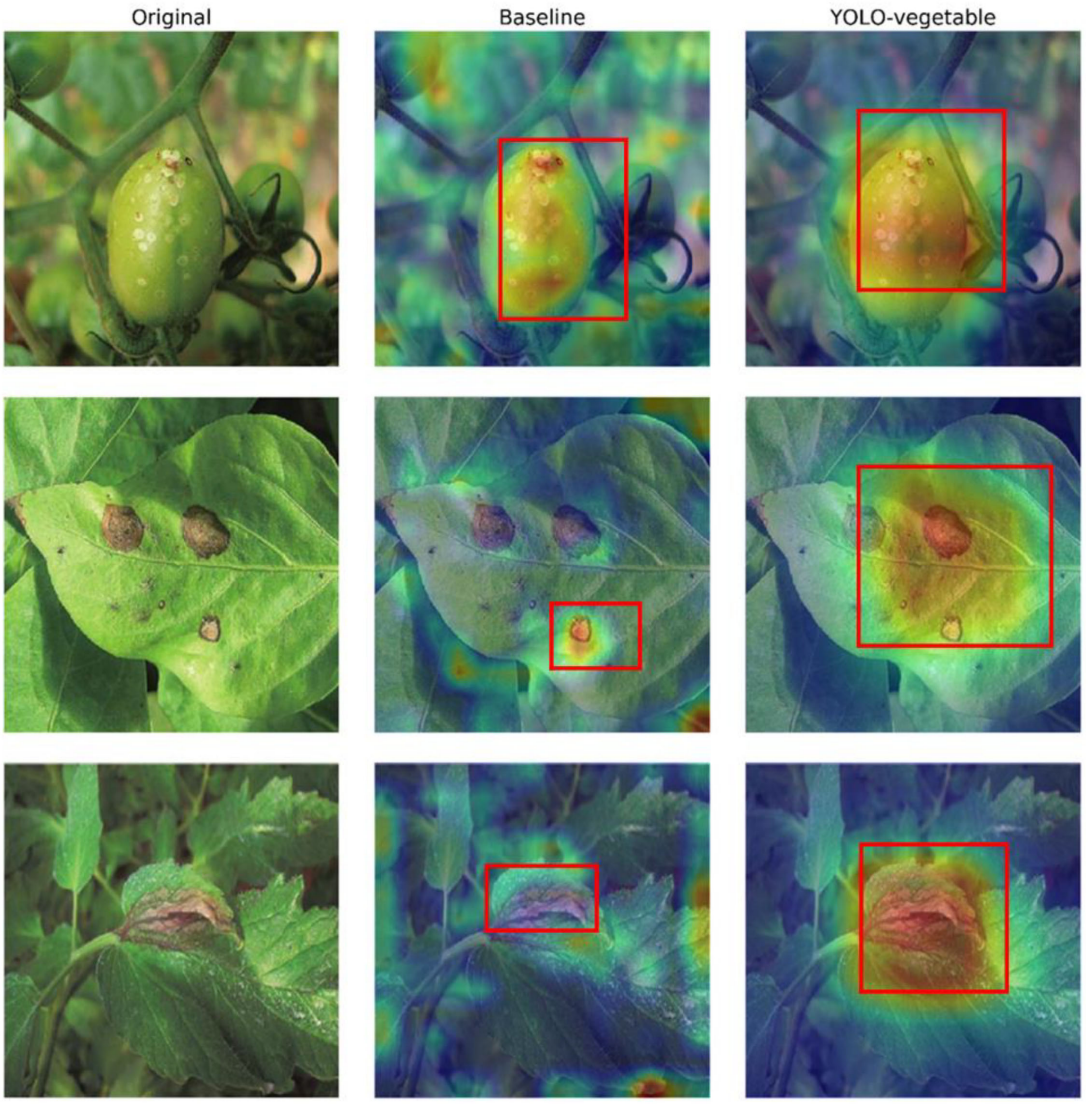
Feature activation maps using Grad-CAM technique.

To better understand the model’s decision-making process, we visualized the feature activation maps using Grad-CAM techniques (**Figure 13**). These visualizations demonstrate that our YOLO-vegetable model correctly focuses on disease-affected regions while effectively filtering out background noise. For smaller lesions, the model exhibits precise localization, confirming the effectiveness of our detail-preserving modules. Comparative analysis of activation maps between the baseline model and YOLO-vegetable reveals distinct differences in feature focus. While the baseline model tends to activate broadly across leaf surfaces with disease-like coloration patterns, our model demonstrates more precise localization specifically on the actual disease lesions. This is particularly evident in the second row of **Figure 13**, where the baseline model shows diffuse activation across multiple spots, while YOLO-vegetable concentrates activation intensity precisely on the primary disease lesions. This improved focus significantly reduces false positives in complex backgrounds with similar color patterns to diseases but different textural features, a common challenge in greenhouse environments with varying light conditions creating shadowing effects that resemble disease symptoms.

### 4.6 Comparative experiments

**Figure 14:**
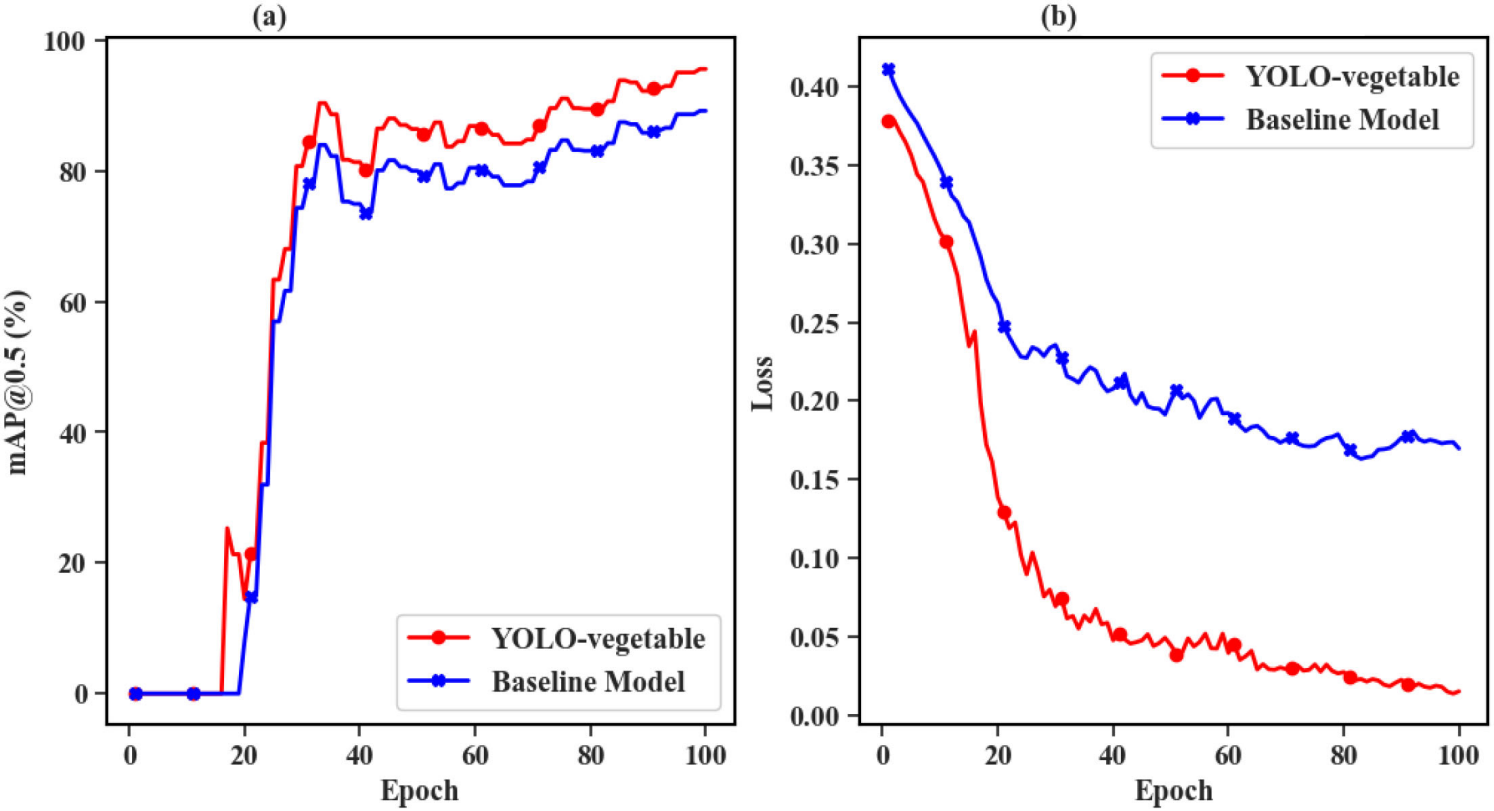
Comparison results between the proposed YOLO-vegetable model and the baseline model during training process. **(a)** mAP@0.5 curves: YOLO-vegetable (red line) achieves faster convergence and higher final performance (95.6%) compared to baseline model (blue line, 89.2%), demonstrating superior learning capability. **(b)** Loss curves: YOLO-vegetable (red line) exhibits lower loss values and more stable convergence behavior compared to baseline model (blue line), indicating more effective optimization and better model training dynamics.

**Figure 14** presents the comparative experimental results between the proposed YOLO-vegetable model and the baseline model during the training process. The left subfigure (a) shows the mAP@0.5 curves of YOLO-vegetable and the baseline model. From the figure, it is evident that the proposed model achieves higher mAP during training and converges faster, ultimately reaching 95.6% mAP, significantly outperforming the baseline model’s 89.2%. The right subfigure (b) displays the Loss curves of YOLO-vegetable and the baseline model. The baseline model exhibits higher Loss values, slower convergence speed, and ultimately higher final Loss values compared to the proposed model. This indicates that the proposed YOLO-vegetable model not only surpasses the baseline model in accuracy but also demonstrates better convergence behavior and lower loss during training.

To comprehensively evaluate the performance of YOLO-vegetable, we conducted extensive comparisons with mainstream object detection models. The experimental results are summarized in **Table 5**. On the same vegetable disease dataset, the proposed model exhibits superior comprehensive performance. In terms of detection accuracy, YOLO-vegetable achieves 95.6% mAP@0.5, significantly exceeding the baseline model YOLOv10n (89.2%) and outperforming other mainstream detection algorithms such as Faster-RCNN (89.6%), SSD (94.2%), and YOLOv5s (93.9%). Notably, the proposed model achieves comparable performance to YOLOv10s (95.5%) while demonstrating superior resource efficiency.

**Table 5:** Performance comparison with state-of-the-art models.

Model	mAP@0.5 (%)	Parameters (M)	FLOPs (G)	Inference Time (ms/frame)
Faster-RCNN	89.6	63.2	370	114.8
SSD	94.2	12.3	63.2	22.2
YOLOv3	77.8	61.8	43.2	18.9
YOLOv5s	93.9	9.1	23.8	17.2
YOLOv8s	92.5	11.2	28.5	19.1
YOLOv10n	89.2	2.2	6.5	15.6
YOLOv10s	94.5	7.2	21.4	24.8
YOLOv11n	91.7	2.5	6.3	15.6
YOLOv11s	94.0	9.4	21.3	21.8
YOLO-vegetable (Ours)	95.6	3.8	14.7	18.6

From the perspective of model complexity, YOLO-vegetable exhibits significant advantages. Compared to Faster-RCNN’s 63.2M parameters, the proposed model requires only 3.8M parameters, reducing storage demands by approximately 94%. In terms of computational efficiency, YOLO-vegetable achieves 14.7 GFLOPs, substantially lower than Faster-RCNN (370.0 GFLOPs) and SSD (63.2 GFLOPs), and also outperforms YOLOv5s (23.8 GFLOPs) and YOLOv8s (28.5 GFLOPs). This marked reduction in computational cost makes the model more suitable for deployment in resource-constrained practical applications.

Regarding real-time performance, YOLO-vegetable achieves an average inference time of 18.6ms per frame, significantly outperforming two-stage detectors such as Faster-RCNN (114.8ms/frame) and single-stage detectors like SSD (22.2ms/frame). Although there is a slight increase compared to the baseline model YOLOv10n (15.6ms/frame), this latency increment is acceptable given the substantial improvement in detection accuracy (from 89.2% to 95.6%). Particularly, compared to YOLOv10s (24.8ms/frame) and YOLOv11s (21.8ms/frame), the proposed model achieves lower inference latency while maintaining comparable detection accuracy, which is crucial for real-time disease monitoring in greenhouse environments.

Through comparisons with various YOLO series variants, it is evident that YOLO-vegetable achieves an optimal balance between performance and lightweight design. Compared to lightweight models such as YOLOv10n (89.2%) and YOLOv11n (91.7%), the proposed model achieves significant accuracy improvements with only moderate increases in parameter count. When compared to YOLOv10s (94.5%) and YOLOv11s (94.0%), it maintains comparable accuracy while substantially reducing model complexity and computational overhead. This balanced performance fully validates the effectiveness of the proposed improvement strategies and provides an efficient and practical solution for vegetable disease detection in real-world applications.

To evaluate the robustness of the proposed model under noisy conditions, we conducted experiments with Gaussian and salt-and-pepper noise. The results demonstrate that YOLO-vegetable maintains high detection accuracy, with mAP@0.5 above 90% in both noise scenarios, highlighting its robustness in real-world applications.

**Figure 15:**
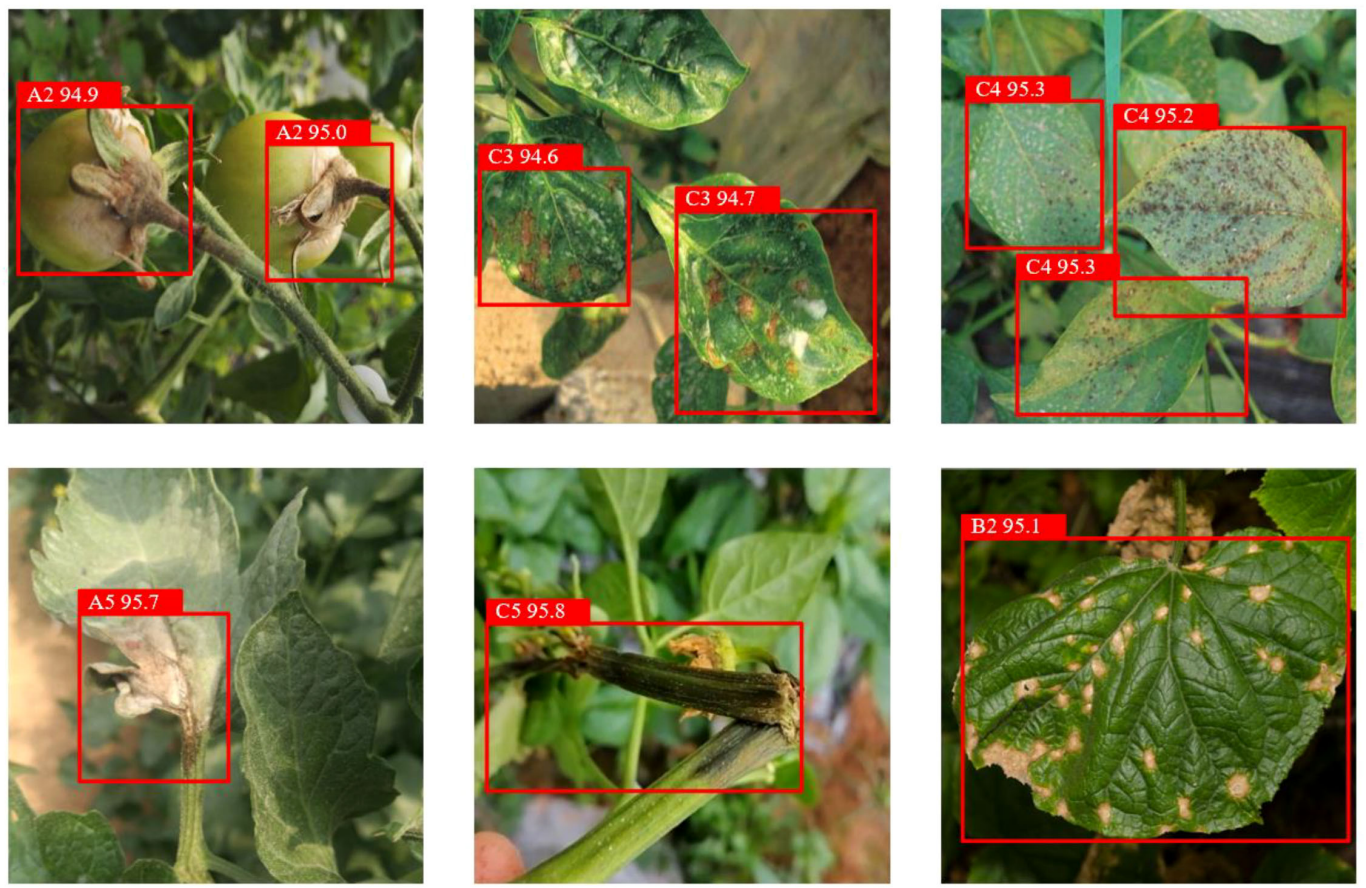
Detection results with bounding boxes.

**Figure 15** presents representative detection results with bounding boxes across various greenhouse scenarios, including different lighting conditions, planting densities, and disease severities. The visualizations demonstrate YOLO-vegetable’s superior detection performance particularly in challenging cases such as partially occluded leaves, early-stage disease symptoms, and complex backgrounds with shadows. Compared to baseline models, our approach shows notably fewer false positives on healthy plant parts with similar color patterns to diseased regions, indicating enhanced feature discrimination capabilities.

### 4.7 Generalization experiments

To validate the generalization capability of the proposed YOLO-vegetable model, a public dataset downloaded from the Baidu PaddlePaddle platform in China was selected for generalization testing. This dataset contains 534 images and corresponding annotation files, exhibiting strong scene diversity and challenges. The download link is: https://aistudio.baidu.com/datasetdetail/292158. Training was conducted under consistent hardware conditions. The experimental results are shown in **Table 6**.

**Table 6:** Generalization experiment results.

Model	Precision (%)	Recall (%)	mAP@0.5 (%)
Baseline	86.5	84.2	85.4
YOLO-vegetable (Ours)	94.8	93.6	94.2

The experimental results demonstrate that YOLO-vegetable exhibits excellent performance advantages on cross-scene datasets. Compared to the baseline model, precision increased by 8.3% (from 86.5% to 94.8%), indicating that the improved model maintains high detection accuracy in unknown scenarios. Recall increased by 9.4% (from 84.2% to 93.6%), proving that the model has a stronger ability to detect diseases. The mean average precision (mAP@0.5) increased by 8.8% (from 85.4% to 94.2%), demonstrating a significant improvement in the model’s overall performance. This comprehensive performance enhancement fully validates the effectiveness of the proposed improvement strategies.

In-depth analysis reveals that the superior generalization performance of YOLO-vegetable is primarily attributed to its enhanced feature representation capability. Through the adaptive detail enhancement mechanism of the ADEConv module, the model can better extract and retain fine-grained features of diseases, enabling accurate recognition across different scenarios. The multi-granularity feature fusion mechanism of the MFLayer allows the model to adaptively handle disease targets of different scales, effectively addressing the issue of target scale variation in cross-scene data. Additionally, the dynamic feature fusion strategy of the IDFNet significantly enhances the model’s adaptability to complex backgrounds, ensuring stable detection performance under varying lighting, angles, and occlusion conditions.

Qualitative analysis shows that YOLO-vegetable exhibits clear advantages in handling complex scenarios (e.g., lighting variations, partial occlusion, complex backgrounds), with both false detection and missed detection rates lower than those of the baseline model. This fully demonstrates that the proposed improvement strategies not only enhance the model’s detection accuracy but also improve its generalization capability and environmental adaptability. The experimental results indicate that the YOLO-vegetable model has excellent generalization performance, maintaining stable detection performance when faced with new, unseen data, laying a technical foundation for the large-scale application of the model in practical agricultural production.

## 5 Conclusions and future work

### 5.1 Conclusions

This study successfully addresses critical challenges in greenhouse vegetable disease detection by developing YOLO-vegetable, an enhanced deep learning architecture that significantly improves detection accuracy while maintaining computational efficiency. Our approach represents a substantial advancement in applying AI technology to support agricultural new quality productive forces.

The key contributions of this work include: (1) innovative architectural designs that preserve fine-grained features while enabling multi-scale detection; (2) comprehensive experimental validation demonstrating superior performance across diverse greenhouse conditions; and (3) practical deployment considerations with optimized parameter efficiency. The proposed method achieves state-of-the-art performance on our comprehensive dataset while maintaining real-time capabilities essential for practical applications.

Experimental results validate the effectiveness of our approach, with significant improvements in detection accuracy and computational efficiency compared to existing methods. The model’s robust performance across different disease types, growth stages, and environmental conditions demonstrates its potential for widespread adoption in intelligent greenhouse systems. This work provides a foundation for advancing precision agriculture through AI-driven disease monitoring and contributes to the development of sustainable agricultural practices.

### 5.2 Future work

Future research directions include: (1) comprehensive cross-regional validation to establish model generalizability across diverse agricultural settings; (2) development of lightweight architectures for edge computing deployment; (3) integration with IoT systems for automated greenhouse monitoring; and (4) extension to additional crop varieties and disease types. Long-term goals focus on creating comprehensive agricultural AI platforms that support large-scale implementation of intelligent disease management systems in modern farming operations.

## Data Availability

The datasets presented in this study can be found in online repositories. The names of the repository/repositories and accession number(s) can be found in the article/**Supplementary Material**.

## References

[B1] Abdalla A. Wheeler T. A. Dever J. Lin Z. Arce J. Guo W.. (2024). Assessing fusarium oxysporum disease severity in cotton using unmanned aerial system images. Biosyst. Eng. 237, 220–231. doi: 10.1016/j.biosystemseng.2023.12.014

[B2] Ali A. M. Słowik A. Hezam I. M. Basset M. A. (2024). Sustainable smart system for vegetables plant disease detection: Four vegetable case studies. Comput. Electron. Agric. 227. doi: 10.1016/j.compag.2024.109672

[B3] Alif M. A. R. Hussain M. (2024). YOLOv1 to YOLOv10: A comprehensive review of YOLO variants and their application in the agricultural domain. arXiv preprint arXiv:2406.10139.

[B4] Bao W. Zhu Z. Hu G. Zhou X. Zhang D. Yang X. (2023). UAV remote sensing detection of tea leaf blight based on DDMA-YOLO. Comput. Electron. Agric. 205. doi: 10.1016/j.compag.2023.107637

[B5] Barbedo J. G. A. (2019). Plant disease identification from individual lesions and spots using deep learning. Biosyst. Eng. 180. doi: 10.1016/j.biosystemseng.2019.02.002

[B6] Bonora A. Bortolotti G. Bresilla K. Grappadelli L. C. Manfrini L.. (2021). A convolutional neural network approach to detecting fruit physiological disorders and maturity in ‘Abbé Fétel’ pears. Biosyst. Eng. 212, 264–272. doi: 10.1016/j.biosystemseng.2021.10.009

[B7] Bouni M. Hssina B. Douzi K. Douzi S.. (2024). Synergistic use of handcrafted and deep learning features for tomato leaf disease classification. Sci. Rep. 14, 26822., PMID: 39500934 10.1038/s41598-024-71225-5 PMC11538303

[B8] Castillo-Girones S. Munera S. Martínez-Sober M. Blasco J. Cubero S. Gómez-Sanchis J. (2025). Artificial neural networks in agriculture, the core of artificial intelligence: what, when, and why. Comput. Electron. Agric. 230, 109938.

[B9] Chang S. Yang G. Cheng J. Feng Z. Fan Z. Ma X.. (2024). Recognition of wheat rusts in a field environment based on improved DenseNet. Biosyst. Eng. 238, 10–21. doi: 10.1016/j.biosystemseng.2023.12.016

[B10] Chen C. F. R. Fan Q. Panda R. (2021). Crossvit: Cross-attention multi-scale vision transformer for image classification. In Proc. IEEE/CVF Int. Conf. Comput. Vision pp, 357–366. doi: 10.1109/ICCV48922.2021.00041

[B11] Chowdhury R. Arko P. S. Ali M. E. Khan M. A. I. Apon S. H. Nowrin F.. (2020). Identification and recognition of rice diseases and pests using convolutional neural networks. Biosyst. Eng. 194.

[B12] Ding M. Xiao B. Codella N. Luo P. Wang J. Yuan L. (2022). Davit: Dual attention vision transformers. In European conference on computer vision (Cham: Springer Nature Switzerland), 74–92.

[B13] Han K. Wang Y. Tian Q. Guo J. Xu C. Xu C. (2020). “Ghostnet: More features from cheap operations,” in Proceedings of the IEEE/CVF conference on computer vision and pattern recognition. 1580–1589.

[B14] Hari P. Singh M. P. (2025). Adaptive knowledge transfer using federated deep learning for plant disease detection. Comput. Electron. Agric. 229, 109720. doi: 10.1016/j.compag.2024.109720

[B15] Hu G. Yin C. Wan M. Zhang Y. Fang Y. (2024). Recognition of diseased plants using multi-spectral fusion. Biosyst. Eng.

[B16] Jian T. Qi H. Chen R. Jiang J. Liang G. Luo X.. (2025). Identification of tomato leaf diseases based on DGP-SNNet. Crop Prot. 187, 106975. doi: 10.1016/j.cropro.2024.106975

[B17] Johri P. Kim S. Dixit K. Sharma P. Kakkar B. Kumar Y.. (2024). Advanced deep transfer learning techniques for efficient detection of cotton plant diseases. Front. Plant Sci. 15. doi: 10.3389/fpls.2024.1441117, PMID: 39759238 PMC11696538

[B18] Kang R. Huang J. Zhou X. Ren N. Sun S. (2024). Toward real scenery: A lightweight tomato growth inspection algorithm for leaf disease detection and fruit counting. Plant phenomics 6. doi: 10.34133/plantphenomics.0174, PMID: 38629080 PMC11018486

[B19] Karantoumanis E. Balafas V. Louta M. Ploskas N. (2024). Real-time disease detection on bean leaves from a small image dataset using data augmentation and deep learning methods. Soft Computing 28. doi: 10.1007/s00500-024-10348-3

[B20] Kumar V. S. Jaganathan M. Viswanathan A. Umamaheswari M. Vignesh J. J. E. R. C. (2023). Rice leaf disease detection based on bidirectional feature attention pyramid network with YOLO v5 model. Environ. Res. Commun. 5, 065014. doi: 10.1088/2515-7620/acdece

[B21] Lin J. Hu G. Chen J. (2024). Mixed data augmentation and osprey search strategy for enhancing YOLO in tomato disease, pest, and weed detection. Expert Syst. With Appl. 264.

[B22] Liu X. Min W. Mei S. Wang L. Jiang S. (2021). Plant disease recognition: A large-scale benchmark dataset and a visual region and loss reweighting approach. IEEE Trans. Image Process. 30. doi: 10.1109/TIP.83, PMID: 33444137

[B23] Liu C. Zhu H. Guo W. Han X. Chen C. Wu H. (2024). EFDet: An efficient detection method for cucumber disease under natural complex environments. Comput. Electron. Agric.

[B24] Mathieu L. Reder M. Siah A. Ducasse A. Langlands-Perry C. Marcel T. C.. (2024). SeptoSympto: a precise image analysis of Septoria tritici blotch disease symptoms using deep learning methods. Plant Methods 20, 18. doi: 10.1186/s13007-024-01136-z, PMID: 38297386 PMC10832182

[B25] Mhala P. Bilandani A. Sharma S. (2025). Enhancing crop productivity with fined-tuned deep convolution neural network for Potato leaf disease detection. Expert Syst. With Appl. 267. doi: 10.1016/j.eswa.2024.126066

[B26] Mo H. Wei L. (2024). Lightweight citrus leaf disease detection model based on ARMS and cross-domain dynamic attention. J. King Saud Univ. - Comput. Inf. Sci. 36. doi: 10.1016/j.jksuci.2024.102133

[B27] Paul N. Sunil G. C. Horvath D. Sun X. (2025). Deep learning for plant disease detection: A comprehensive review of technologies, challenges, and future directions. Comput. Electron. Agric. 229.

[B28] Qing J. Deng X. Lan Y. Li Z. (2023). GPT-aided diagnosis on agricultural image based on a new light YOLOPC. Comput. Electron. Agric. 213. doi: 10.1016/j.compag.2023.108168

[B29] Sun C. Li Y. Song Z. Liu Q. Si H. Yang Y.. (2025). Research on tomato disease image recognition method based on DeiT. Eur. J. Agron. 162. doi: 10.1016/j.eja.2024.127400

[B30] Tan M. Pang R. Le Q. V. (2020). Efficientdet: Scalable and efficient object detection. Proc. IEEE/CVF Conf. Comput. Vision Pattern recognition, 10781–10790. doi: 10.1109/CVPR42600.2020

[B31] Tian L. Zhang H. Liu B. Zhang J. Duan N. Yuan A.. (2022). VMF-SSD: A novel V-space based multi-scale feature fusion SSD for apple leaf disease detection. IEEE/ACM Trans. Comput. Biol. Bioinf. doi: 10.1109/TCBB.2022.3229114, PMID: 37015544

[B32] Toda Y. Okura F. (2019). How convolutional neural networks diagnose plant disease. Plant Phenomics. doi: 10.34133/2019/9237136, PMID: 33313540 PMC7706313

[B33] Upadhyay A. Chandel N. S. Singh K. P. Chakraborty S. K. Nandede B. M. Kumar M.. (2025). Deep learning and computer vision in plant disease detection: a comprehensive review of techniques, models, and trends in precision agriculture. Artif. Intell. Rev. 58, 1–64. doi: 10.1007/s10462-024-11100-x

[B34] Vásconez J. P. Vásconez I. N. Moya V. Calderón-Díaz M. J. Valenzuela M. Besoain X.. (2024). Deep learning-based classification of visual symptoms of bacterial wilt disease caused by Ralstonia solanacearum in tomato plants. Comput. Electron. Agric. 227, 109617. doi: 10.1016/j.compag.2024.109617

[B35] Wang A. Chen H. Liu L. Chen K. Lin Z. Han J.. (2024). Yolov10: Real-time end-to-end object detection. arXiv preprint arXiv:2405.14458.

[B36] Wang H. He M. Zhu M. Liu G. (2024). WCG-VMamba: A multi-modal classification model for corn disease. Comput. Electron. Agric. 230.

[B37] Wójcik Gront E. Zieniuk B. Pawełkowicz M.. (2024). Harnessing AI-powered genomic research for sustainable crop improvement. Agriculture 14, 2299. doi: 10.3390/agriculture14122299

[B38] Xu Y. Chen Q. Kong S. Xing L. Wang Q. Cong X.. (2022). Real-time object detection method of melon leaf diseases under complex background in greenhouse. J. Real-Time Image Process. 19. doi: 10.1007/s11554-022-01239-7

[B39] Yan K. Guo X. Ji Z. Zhou X. (2024). Deep transfer learning for cross-species plant disease diagnosis adapting mixed subdomains. IEEE/ACM Trans. Comput. Biol. Bioinf., PMID: 34914593 10.1109/TCBB.2021.3135882

[B40] Yang N. Chang K. Dong S. Tang J. Wang A. (2024). Plant disease detection with vision-language fusion framework. Comput. Electron. Agriculture.

[B41] Ye R. Shao G. Yang Z. Sun Y. Gao Q. Li T. (2024). Detection model of tea disease severity under low light intensity based on YOLOv8 and enlightengan. Plants 13. doi: 10.3390/plants13101377, PMID: 38794447 PMC11125842

[B42] Zhang D. Luo H. S. Cheng T. Li W. F. Zhou X. G. Gu C. Y.. (2023). Enhancing wheat Fusarium head blight detection using rotation Yolo wheat detection network. Comput. Electron. Agric. 211, 107968.

[B43] Zhang D. Zhang W. Cheng T. Zhou X. Yan Z. Wu Y.. (2024). Detection of wheat scab fungus spores utilizing the Yolov5-ECA-ASFF network structure. Comput. Electron. Agric. 210.

[B44] Zhao Y. Chen Z. Gao X. Song W. Xiong Q. Hu J.. (2025). Plant disease detection using generated leaves based on doubleGAN. IEEE/ACM Trans. Comput. Biol. Bioinf., PMID: 33534712 10.1109/TCBB.2021.3056683

[B45] Zhou L. Xiao Q. Taha M. F. Xu C. Zhang C. (2023). Phenotypic analysis of diseased plant leaves using supervised and weakly supervised deep learning. Plant Phenomics 5. 10.34133/plantphenomics.0022 PMC10076051 37040509

